# Situating language register across the ages, languages, modalities, and cultural aspects: Evidence from complementary methods

**DOI:** 10.3389/fpsyg.2022.964658

**Published:** 2023-01-04

**Authors:** Valentina N. Pescuma, Dina Serova, Julia Lukassek, Antje Sauermann, Roland Schäfer, Aria Adli, Felix Bildhauer, Markus Egg, Kristina Hülk, Aine Ito, Stefanie Jannedy, Valia Kordoni, Milena Kuehnast, Silvia Kutscher, Robert Lange, Nico Lehmann, Mingya Liu, Beate Lütke, Katja Maquate, Christine Mooshammer, Vahid Mortezapour, Stefan Müller, Muriel Norde, Elizabeth Pankratz, Angela G. Patarroyo, Ana-Maria Pleşca, Camilo R. Ronderos, Stephanie Rotter, Uli Sauerland, Gohar Schnelle, Britta Schulte, Gediminas Schüppenhauer, Bianca Maria Sell, Stephanie Solt, Megumi Terada, Dimitra Tsiapou, Elisabeth Verhoeven, Melanie Weirich, Heike Wiese, Kathy Zaruba, Lars Erik Zeige, Anke Lüdeling, Pia Knoeferle

**Affiliations:** ^1^Department of German Studies and Linguistics, Humboldt-Universität zu Berlin, Berlin, Germany; ^2^Institute of Archaeology, Humboldt-Universität zu Berlin, Berlin, Germany; ^3^Institute of Romance Studies, Universität zu Köln, Cologne, Germany; ^4^Department of English and American Studies, Humboldt-Universität zu Berlin, Berlin, Germany; ^5^Department of English, Linguistics and Theatre Studies, National University of Singapore, Singapore, Singapore; ^6^Leibniz-Centre General Linguistics, Berlin, Germany; ^7^Department for Northern European Studies, Humboldt-Universität zu Berlin, Berlin, Germany; ^8^Centre for Language Evolution, University of Edinburgh, Edinburgh, United Kingdom; ^9^Department of Philosophy, Classics, History of Art and Ideas, University of Oslo, Oslo, Norway; ^10^Institute for Germanic Linguistics, Friedrich-Schiller-Universität Jena, Jena, Germany; ^11^Berlin School of Mind and Brain, Humboldt-Universität zu Berlin, Berlin, Germany; ^12^Einstein Center for Neurosciences Berlin-Charité, Berlin, Germany

**Keywords:** language register, situational context, behavioral methods, corpus methods, register markers, language variation

## Abstract

In the present review paper by members of the collaborative research center “Register: Language Users' Knowledge of Situational-Functional Variation” (CRC 1412), we assess the pervasiveness of register phenomena across different time periods, languages, modalities, and cultures. We define “register” as recurring variation in language use depending on the function of language and on the social situation. Informed by rich data, we aim to better understand and model the knowledge involved in situation- and function-based use of language register. In order to achieve this goal, we are using complementary methods and measures. In the review, we start by clarifying the concept of “register”, by reviewing the state of the art, and by setting out our methods and modeling goals. Against this background, we discuss three key challenges, two at the methodological level and one at the theoretical level: (1) To better uncover registers in text and spoken corpora, we propose changes to established analytical approaches. (2) To tease apart between-subject variability from the linguistic variability at issue (intra-individual situation-based register variability), we use within-subject designs and the modeling of individuals' social, language, and educational background. (3) We highlight a gap in cognitive modeling, viz. modeling the mental representations of register (processing), and present our first attempts at filling this gap. We argue that the targeted use of multiple complementary methods and measures supports investigating the pervasiveness of register phenomena and yields comprehensive insights into the cross-methodological robustness of register-related language variability. These comprehensive insights in turn provide a solid foundation for associated cognitive modeling.

## 1. Introduction

### 1.1. Defining and modeling linguistic (register) variability

It has been widely observed that speakers vary the linguistic means applied in communication depending on the situational-functional context (e.g., whether language is used to narrate or instruct and whether the situation is formal or not). Linguistic variation in this sense pertains to the lexicon, phonetic realization, morphosyntax, and their integration into more complex meaning including discourse structure. It occurs in spoken, signed, and written, modern and historical languages all over the world. This phenomenon has been investigated and modeled under the umbrella term of “register variation”. In the CRC 1412^1^, we define registers as *intra*-individual variation, that is, the conventionalized and recurrent linguistic patterns of (individuals in) a speech community depending on the situational-functional context. The goal of the research center is to develop an empirically-motivated model of register knowledge underpinning linguistic behavior in situational-functional contexts.

Central to the study of language in context is the assumption that we can observe behavior and that we can develop, based on a series of observations, models of the mental representations involved in context-based language processing and use. Context encompasses the extra-linguistic situation in which language is produced and processed. This includes time and place of the communication, the number and identity of participants (their age, gender, ethnicity, status, education, and social role, among others). As an example of this kind of variation, consider the usage of forms of address in different situational contexts. In the principal's office as a highly formal situation, students may be less likely to address their interlocutor as *dude* or *bro* than in a local pub where these expressions would be in line with the informal situation and used more often. While formality and similar parameters can be conveyed via the extra-linguistic context (e.g., a situation), our notion of context also includes linguistic context (e.g., a description of a situation).^2^

Following this working definition, we briefly situate the present investigation of register in the context of prior research and then outline the goals of the present paper. By its very nature, register is associated with extra-linguistic and linguistic contexts; both have been theoretically and/or methodologically conceptualized by different research traditions. Based on ethnological/anthropological context models (Malinowski, 1923, 1935; Firth, 1957), the term “register” was coined by Reid (1956), as reviewed in Goulart et al. (2020). It is a key notion in the grammar modeling of Systemic Functional Linguistics (SFL) (Halliday, 1978; Neumann, 2013; Halliday and Matthiessen, 2014; Hasan, 2014), in which communicative context plays a constitutive role (Bowcher, 2020). This language theory mainly focuses on the modeling of the extra-linguistic component. Starting with the Field-Tenor-Mode (FTM) model, various paradigms were developed between 1978 and 2014^3^.

The SFL perspective on registers aims at developing a functional theory of language use. In addition it has inspired empirical studies and applications with mainly qualitative (Hasan, 2015) but also some quantitative approaches (Neumann, 2013; Matthiessen, 2015)^4^. The basic FTM model can serve as a starting point for an empirical operationalization of context. Depending on the languages, historical periods, nature of the data, transmission situation, and methods applied, modifications and additions to the FTM model became necessary (e.g., a more explicit modeling of communicative functions via “Field”; the internal restructuring of the sub-dimension “Tenor”^5^ and an expansion of the communication channel, “Mode”).

Parallel to the development of extra-linguistic contextual models in SFL, Labov (1966/2006), as a pioneer of the sociolinguistic variationist paradigm, investigated “contextual styles” as an expression of language use depending on formality (i.e., attention paid to speech). As part of this endeavor, studies examined register effects depending on formality or modality (spoken vs. written) of context, or depending on specific genres (e.g., personal letters, novels, newspaper articles). Building on this earlier research, Biber (1986) laid the foundation for a quantitative contrastive characterization of registers by means of Multidimensional Analysis. Indeed, seminal contributions on register come from the text-linguistic and variationist-linguistic literature (Goulart et al., 2020 for a comprehensive review). From this part of the field, we gain the insight that characteristics of, and the function of language in, a situation can prompt language users to adjust their register. Evidence for this has come from the detailed analysis of individual linguistic features in the production of speech and written text (including electronic, literary, and historical sources). What has also been highlighted is that recent research has shifted attention to specialized written and spoken domains; for these, statistical methods like multidimensional analysis permitted a comprehensive analysis of registers (see also Section 1.3.1 for a review).

Against this state of the art, we present an overview of the investigation into register phenomena within the collaborative research center (CRC) “Register 1412”. In doing so, we illustrate the pervasiveness of register effects across the ages, languages, modalities, and cultures, extending findings in the literature. We present the methodological backbone of this investigation (Section 1.2), changes to extant methods and discuss both methodological (Sections 1.3.1 and 1.3.2) and theoretical (Section 1.3.3) challenges. The goal is to use the insights from the corpus and experimental investigations (Sections 2 and 3) for modeling (the linguistic representations and processes implicated in) register variation. We present the results and methods of our systematic multi-project investigation of register and add to first attempts at modeling the mental representations related to register knowledge (Section 4, see also Keller, 2021, p. 81, 83, 98ff.). We hope to subsequently be able to integrate the findings of the current CRC funding phase into modeling register knowledge, covering its change, learning, perception, comprehension, and production across different social strata, ages, cultures, and languages.

### 1.2. Overview of the complementary methods and measures

We next provide an overview of the corpus and experimental methods employed and highlight how we combine these in the investigation of register. Compiling and using specific corpora in Berlin and further afield, we examine how characteristics of a situation (e.g., its formality, or written or oral modality), or of a text (e.g., its purpose), or of social relations between interlocutors (e.g., power inequality of the interlocutors) shape the language register. We hypothesize that these aspects of context co-occur with differences in which “variants” of sounds, words, and grammar are used. Variants in this sense are conceptualized as concrete instantiations of an abstract variable. For example, if a student at a German university asks a clerk in the enrolment office of the university for a specific administrative form, he or she would likely refer to it with the variant *Formular* (“form”). By contrast, in an informal conversation with a friend, the same document might be called *Wisch* (“scrap of paper”). Comparing the use of such variants of a variable (here on the lexical level) influenced by the social requirements of a communicative situation, we can infer aspects of the associated mental representations and based on that model register knowledge.

The terms of “variable” and its “variants” are derived from Labov's modeling of variation (Labov, 1966/2006, 2001; Bayley, 2013; Tagliamonte, 2016). The fundamental idea is that many cases of variation can be understood as a single abstract variable which can be expressed by different functionally equivalent concrete variants. We acknowledge the problems this conceptualization of variables entails when it comes to the identification and determination of variants (e.g., whether functional equivalence requires synonymy). However, we consider the extension of this concept beyond the phonological level as in Labov to be helpful in investigating the pervasiveness of register variation. It enables us to examine bundles of related linguistic phenomena with respect to their distribution over registers. For instance, we can better understand how complex noun phrases are constructed in, and distributed across, specific registers if we understand different types of attribution to a noun as variants of the variable “nominal modification” (see, e.g., Biber, 2019b; Egbert et al., 2020, for a relevant discussion of text-linguistic and variationist perspectives). This conceptualization of variables paves the way for a systematic comparison of registers.

Experimental data collection both in the field and in laboratories can complement corpus-based methods (see, e.g., Gilquin and Gries, 2009; Keller, 2021, for a related research). For instance, if we encountered *Wisch* (“scrap of paper”) to refer to a legal document in a formal setting, the situation and hence the language register with its level of formality would mismatch. If this sort of register knowledge is part of the language user's repertoire and can be manipulated in a systematic fashion, then in an experiment, we should see lower acceptability ratings (Fanselow and Frisch, 2006) and perhaps slower responses for mismatches than matches. During real-time processing, we should see reading-time increases in the mismatching, formal (vs. matching, informal) situation at “scrap of paper”. From these behavioral reactions to mismatches (vs. matches), we can infer that “scrap of paper” (and its underlying representation) was unexpected, eliciting processing difficulty. From this we can infer the initially expected mental representation. Via such informal^6^ “linking hypotheses” (Just and Carpenter, 1976; Fanselow and Frisch, 2006), a wide range of behavioral observations has provided insight into the mental representations implicated in language processing and use in context (Hale, 2003; Levy, 2008; Crocker et al., 2012; Venhuizen et al., 2018, on formal linking hypotheses).

Corpus and experimental data complement each other and, in doing so, they provide a solid foundation for modeling register knowledge. Corpus data when compiled from published text sources or spontaneous speech data can be viewed as “naturalistic” in so far as its producers are not influenced by a controlled experimental manipulation. However, the situational context of corpus data can be controlled to a limited degree only and may, as a result, include more random variability than a more controlled experimental setup. Our methodological portfolio combines the controlled setup of lab experiments with the more naturalistic usage conditions in corpus data. The measures we collect encompass both offline and online measures. Offline measures like binary responses, acceptability or plausibility ratings, or verbal production data provide good insight into overt choices in linguistic behavior. More covert responses can be uncovered via measurement of visual attention during reading or spoken language processing. Using measures like acceptability or appropriateness ratings (e.g., Schütze, 2006, 2010; Sprouse, 2011), matched-guise (Lambert et al., 1965), and open-guise responses (Soukup, 2013), eye-tracking during reading (Rayner, 1998), or spoken comprehension (Huettig et al., 2011), we can gain insight into how register is processed offline and in real time. The analysis of linguistic features and their correlation with extra-linguistic factors (e.g., distance of interlocutors or social relationships) can permit further inferences on register variation in natural extra-linguistic and social contexts. Jointly these measures and methods provide a multi-faceted insight into register knowledge, and a solid foundation for associated cognitive models. First steps on combining corpus and experimental methods are reported in Keller (2021). This Ph.D. thesis presents a targeted combination of corpus research on register with lexical-level priming in a lexical-decision task and also offers first modeling of lexical representations (e.g., p. 83, Figure 2.1 in Keller, 2021). Additional corpus and psycholinguistic research, for instance, examined the effects of multi-word-sequence frequency on phrasal lexical decision in spoken, fiction, news, and academic registers for both native and non-native language users. Results revealed faster phrasal decision times for more frequent sequences in three out of the four registers and for both native and non-native language users (Kerz et al., 2020). Apart from these isolated attempts, however, cognitive modeling of mental representations and processes implicated in register variation seems lacking.

Going beyond individual corpus and experimental studies, the present projects as part of a collaborative research center in their sum combine corpus analysis based on annotation and statistical methods with a range of experimental methods (elicited production, psychological tests, newspaper correction, open and matched guise, rating and eye-tracking, see the overview in Table 1 for more information). From these combined results, we can, for instance, infer that formality influences production across a range of distinct situations and language levels, suggesting its effects are truly robust; we can further see that it influences also how participants perceive and rate stimuli and that it even rapidly influences language comprehension. For cognitive modeling, these insights suggest that formality is an important aspect of the implicated mental representations; that the relevant mental representations are available in both production and comprehension; and that such marking in mental representations must be accessible within a few hundred milliseconds (for more information on cognitive and Bayesian modeling see Section 4). If we relied on isolated studies, then we could speak less convincingly to the robustness, pervasiveness, and incrementality of register variation/effects than we can now, by drawing on complementary methods, measures and data from different ages, languages, and cultures in the collaborative research center 1412.

**Table 1:** Overview of the projects, their methods, languages, and results in the CRC 1412 Register.

**Methodo- logical approach and focus**	**Project**	**Methods employed**	**Research questions/aims**	**Measures**	**Independent/Predictor variables**	**Expected/Preliminary results**	**Languages under investigation**	**General themes (recurring across different projects)**
	A05	Elicited production	How does context influence the production of time expressions?	Precise vs. imprecise numerical expressions	Formal (police) vs. informal (party) contexts	No explicit results reported (likely, relation between formal context and precise time expression).	German	Social roles and relations. Other context-related factors. Lexical variants. Non-linguistic realizations of register. Social meaning/inferences. Mental representations/models.
	A06	Elicited production, repeated measures	How does formality (acquaintance, location) influence language production?	Spoken conversation (no specific variables mentioned)	Formality, acquaintance/familiarity, location	Register differences between situation and cultures/languages (German vs. Persian vs. Yucatec Mayan) expected.	German, Persian, Yucatec Mayan	Social roles and relations. Other context-related factors.
Empirical: Production	C02	Elicited production, Zoom, repeated measures	How does formality (conveyed by visual appearance and status of the addressees, and conversation topic) affect language production (as assessed by phonetic variables)?	Phonetic variables (f0, vowel realization)	Formality (modified by conversation topic), visual appearance of interlocutor, status (boss vs. new neighbor) - formal vs. less formal	Preliminary results: more variable and higher f0, and more dispersed vowels in formal context.	German	Social roles and relations. Other context-related factors. Pragmatic/Rhetorical devices.
	C04	Written production (academic text written by second-language learners of German)	Do multiple registers coexist within one text? What marks the shift between sequences within one text?	Structural annotation	Dependency and morphological information	Presence of multiple registers within one and the same text type.	German (advanced language learners)	Other context-related factors. Grammatical phenomena.
	C05	Elicited production, language and psychological tests	How does formality influence register production?	Test battery, written text production (lexical features, syntactic features)	Text production: formal context/academic register vs. informal context/non-academic register, acquaintance of addressee (informal context)	Higher register flexibility (differences between contexts) in informal situation, reflecting adaptation to institutional setting and addressee.	German	Social roles and relations. Other context-related factors.
Empirical: Production	C06	Elicited production, Zoom	How do task and addressee influence phonetic realizations in non-native addressee registers (NNAR)?	Phonetic variables (e.g., speech rate/tempo, pauses)	Task, addressee (native, non-native speaker of German)	Preliminary results: less filled pauses and reduced speech rate when speaking to non-native speaker (NNAR).	German (native vs. non-native)	Social roles and relations. Other context-related factors.
	C07	Elicited production (LangSit method), repeated measures, corpus analysis	Collection of comparable texts differing in register (formality) and mode, for the creation of a corpus.	Spoken and written text production	Mode (written/spoken), context/addressee (teacher, friend)	Text should differ depending on context and mode; no results yet: data/corpus to be analyzed in a second step.	German (in a multilingual setting, Namibia)	Social roles and relations. Grammatical phenomena. Lexical variants. Social meaning/inferences.
Empirical: Comprehension, Perception	A05	Matched guise method	To what extent do linguistic form (precision) and context contribute to social meaning (evaluation of the speaker)?	Ratings related to personality attributes (e.g., solidarity, status)	Precision of the linguistic form (*49, 50, about* *50 minutes*), context (casual, relationship-building, persuasive, for-the-record)	Precise forms: higher ratings with respect to competence, approximate forms receive higher ratings on solidarity, but later on anti-solidarity,	German	Social roles and relations. Other context-related factors. Lexical variants. Non-linguistic realizations of register. Social meaning/inferences. Mental representations/models.
	A07	Rating study	How are different forms of negation (negative concord, pleonastic negation, NPI) restricted to register in British and American English?	Appropriateness ratings	Formality (informal-formal), linguistic form (negative concord, pleonastic negation, NPI), American vs. British English	Negative concord less appropriate in formal than informal context; no difference for other forms.	American English, British English	Grammatical phenomena. Other context-related factors.
Empirical: Comprehension, Perception	C03	Eye-tracking	Does formality-register congruence affect real-time sentence processing?	Reading times, fixation proportions	Context formality-register congruence; verb-argument agreement.	Formality-register congruence effects on reading in a pilot study (longer reading times for mismatching verbs).	German	Other context-related factors. Lexical variants. Mental representations/models.
	C03	Rating studies	What is the degree of perceived formality of different variants (used as stimuli), also in relation to linguistic and educational background? Norming for stimulus selection.	Formality ratings of words (nouns, verbs) and sentences, on a scale from 0 (very informal) to 50 (very formal)	Formality, as conveyed by: 1. higher and lower register of target words (nouns and verbs); 2. formality of the situation described in sentences.	Reliable differences in formality ratings of formal vs. informal variants. Some effects of linguistic and educational background.	German	Other context-related factors. Lexical variants. Mental representations/models.
	C07	Newspaper correction	Are lexical and grammatical contact-linguistic features accepted in a formal written register?	Identification and correction	Lexical and grammatical contact-linguistic features	Higher correct rates for lexical items (higher salience)	German (in a multilingual setting, Namibia)	Social roles and relations. Grammatical phenomena. Lexical variants. Social meaning/inferences. Mental representations/models.
Empirical: Comprehension, Perception	C07	Open guise method	To what extent are lexical and grammatical contact-linguistic features involved in register identification (formal and informal)? How are the speakers of texts including such features assessed?	Formal/informal register; ratings with respect to personality traits	Lexical and grammatical contact-linguistic features	Lexical variables more often likely to mark informal register than grammatical; speakers using lexical features assessed as less competent but more humorous.	German (in a multilingual setting, Namibia)	Social roles and relations. Grammatical phenomena. Lexical variants. Social meaning/inferences. Mental representations/models.
	A01	Corpus analysis/ annotation	Identify factors influencing metaphor use in different registers.	Frequency of different types of metaphors.	Text type/domain (e.g., sermons, Parliament speeches, light fiction); SFL-register features (literacy/orality, persuasiveness), metaphor features (form, conventionalization, content)	Interdependence of metaphors and register: non-conventional + extended metaphors in persuasive registers (Parliament speeches, sermons), few metaphors in fiction.	German	Pragmatic/Rhetorical devices. Other context-related factors.
Corpus-based, Computational	A04	Corpus analysis method	Method (based on LDA, Latent Dirichlet Analysis) for uncovering unknown sets of registers.	Number of potential registers with probabilities of associated document and grammatical categories.	Document types (law, stories), grammatical features (e.g., part of speech, syntactic dependencies)	Example extraction which illustrates the method in the text, application of methods to data within the CRC.	German	Other context-related factors. Grammatical phenomena.
	A05	Corpus analysis/ annotation	Validation of SOLT-measure with respect to formality and other register dimensions.	SOLT measure (word frequency according to dictionary vs. subtitle corpus).	Degree of formality of an expression	SOLT-value correlates with degree of formality according to dictionary.	German	Social roles and relations. Other context-related factors. Lexical variants. Non-linguistic realizations of register.
								Social meaning/inferences. Mental representations/models.
	A06	Corpus analysis/ annotation	Impact of register features on referential choice and RLD.	Occurrence of right-dislocation and referential choice in German and Persian.	Situational-functional criteria (media, speaker relation, domain, size)	Correlation between referential choice, speaker relation and domain/public in German.	German, Persian	Social roles and relations. Other context-related factors.
Corpus-based, Computational	B03	Corpus analysis/ annotation	How do linguistic and graphic features mark registers within and across Egyptian texts?	Occurrence/positioning and function of elements	Linguistic features, graphic features (layout, visual salience, color, typography); social role; social semiotic/SFL-features	Picture/gesture differences in addressing higher-rank and lower-rank people.	Ancient Egyptian	Social roles and relations.
	B04	Corpus analysis/ annotation	Identify situational factors influencing language use (Old High German and Old Swedish in Birgitta and Notker).	Frequency of linguistic features.	Social role and relationship (interlocutors), features related to instruction (e.g., imperative, modal verbs)	Linguistic features and situational factors indicators of register-sensitive behavior: social role differences influence the use of subjectives and modal verbs.	Old High German, Old Swedish	Social roles and relations. Grammatical phenomena. Pragmatic/Rhetorical devices.
	C04	Structural annotation	Development of methods to identify multiple registers occurring in one text.	Text sequences in which different register variants coexist.	Different layers of structural annotation, including dependency and morphological information	Presence of multiple registers within one and the same text type.	German (advanced language learners)	Other context-related factors. Grammatical phenomena.

### 1.3. Methodological challenges

The advantages of combining methods and studies notwithstanding, it seems advisable to discuss methodological choices and pay attention to potential challenges as we examine increasingly subtle relations between aspects of the context and language variability (like choices of standard or colloquial register).

#### 1.3.1. Uncovering register in corpora

We expect register distinctions to be observable in corpora of written and spoken language, provided the corpora contain language use from a range of situational and functional contexts^7^. Register differences can be captured by comparing frequency distributions of specific linguistic characteristics. This challenge has been taken up by two different methodological perspectives: variationism with the concept of variables and their variants at the center of the theory (Labov, 1966/2006, 1978, 2001) and Multi-Dimensional Analysis (MDA; e.g., Biber, 1988, 1989, 2009b) combining bundles of linguistic features to yield register descriptions. We adopt some of the central insights of both approaches but also address potential challenges. One important methodological issue in the meaningful interpretation of frequency counts from corpus studies is how individual variants are grouped in variables (what is counted and what the meaning of a variable is, see Biber, 2012; Lüdeling, 2017, for discussion). Added challenges, especially for historical texts, concern the reconstruction of information required to determine a register (i.e., the identification of relevant variables as well as the situational and functional context, see Kytö, 2019). For instance, oral registers are accessible for historical language stages or dead languages only to a limited extent since users cannot be consulted and little to no recordings concerning the extra-linguistic setting are available (but see work on sociolinguistic variation in early modern English, e.g., Nevalainen and Raumolin-Brunberg, 1989; Nevalainen, 1999). As a consequence, a crucial part of the register spectrum cannot be investigated.

Within the variationist implementation of frequency counts, a central challenge is to identify variables and their variants (via annotations). In order to investigate the usage of a variable in a corpus, we must decide what sort of information associated with this variable can and should be annotated. We could annotate variables and variants in a Labovian variationist framework (Labov, 1966/2006, 1978, 2001), but that is not without problems: First, the theoretical definition of a variable and its variants is by no means obvious. Second, the annotation of such variants in a corpus is notoriously problematic. Since variables are—by necessity—abstract and functional, variants cannot be found by merely looking at form (Lüdeling, 2017; see Section 2.3 for discussion). Furthermore, variables can be distinguished as being categorical or continuous. These challenges entail a series of methodological and conceptual problems some of which we will highlight in the ensuing sections.

In MDA studies in the tradition of Biber, frequency counts have a crucial status in the characterization of registers. Biber (1989, p. 5f.) assumes that a “folk-typology of genres” (later called “registers”; Biber, 2009b, p. 823) can be “defined and distinguished on the basis of systematic non-linguistic criteria”, and that they “correspond directly to the text distinctions recognized by mature adult speakers, reflecting differences in external format and situations of use” (Biber, 1989, p. 39). These text distinctions are assumed to correspond to registers, which manifest themselves as categories such as editorials, personal letters, broadcasts, etc.^8^. In MDA, researchers consider corpus documents that have been pre-assigned to such register categories and count the occurrence frequencies of typically 50–100 linguistic categories (called “features”) such as pronouns, modal verbs, types of adverbials, and relative clauses in those documents. These occurrence frequencies produce a feature set, the dimensionality of which is then reduced using a statistical method (typically factor analysis). Registers are analyzed with quantitative factor scores derived from statistical co-occurrence of normalized counts (across texts) and these counts are averaged within register categories (Biber, 2019a, for a recent review). The resulting feature dimensions are functionally interpreted by way of introspection (Biber, 1988, p. 64). More recent theorizing has extended earlier analyses by conceptualizing “variation among texts and registers in a continuous (quantitative) situational space” (Biber, 2020, p. 581). Although MDA is a well-established method for analyzing registers in corpora via frequency distributions, we follow different, innovative routes: First, we do not rely on an established list of registers. Second, we use methods which restrict our options for tweaking the hyperparameters of the model (i.e., the parameters explicitly set by the researcher to tweak the estimation process, as opposed to the parameters actually estimated by the algorithm) (see Section 2). Third, in the collaborative research center we go beyond the categorical variants and frequency measures of occurrences by also analyzing quantitative acoustic measures on the production side and eye-tracking measures on the processing side.

#### 1.3.2. Replicability: The tension between intra- and inter-individual variability

Another important challenge is that we are researching linguistic behavior for which inter-individual variability in linguistic knowledge and behavior is likely high. Such high variability could lead to challenges in reliably observing register differences from language behavior across individuals in both contemporary and historical language use. In historical linguistics, texts are often scarce, vary considerably in length and are unevenly distributed in time, space, and across genres. Individual authors are often unknown and their social-cultural context is lost. Variability between individual text witnesses^9^ must be considered and is addressed by consulting established grammars, dictionaries, historical, and text-critical research when interpreting quantitative results (Jenset and McGillivray, 2017, p. 37ff; Rissanen, 2009, p. 64–66).

For experimental research, we control inter-individual variability through design, random sampling, and extensive pre-testing and piloting. In non-experimental data, by contrast, we must be aware of the potential co-presence of diatopic and diastratic influence (Coseriu, 1980; Koch and Oesterreicher, 1985) in register-sensitive linguistic expressions. First, let us consider an instance of diatopic variation: A colloquial variant of standard German *Fü*β*e* (“feet”) is *Mauken*. One could expect the use of the colloquial variant in informal and of the standard in more formal situations. But the informal variant in this example is not known in all areas of Germany. Also, in specific dialects the variant *Mauken* can come with a negative semantic connotation^10^.

Second, diastratic variation could also influence register variability/effects. People from diverse socio-economic backgrounds can have different understandings of formal and informal registers. This results in associating differing variants with presumably one and the same register. Associations of this kind could pertain to all linguistic levels. To illustrate, consider an example for lexical variants: Person A draws on rather standard-like expressions for her colloquial register, thus using variants like German *speisen* or *tafeln* (“dine”) in formal situations and *essen* (“eat”) in informal ones. By contrast, person B might apply different variants in the same settings. She might use *essen* (“eat”) as formal and *futtern* (“nosh”) as colloquial variant. A and B would employ both registers, but these variants would feature different formality values in their respective registers.

For research on situation-dependent language use within an individual, high inter-individual variability in register-related aspects of language may seem negligible. But given the socially interactive nature of communication, this sort of variability across individuals could lead to a lack of common ground in register use and to disruptions in the processing between individuals (Clark, 1998; Pickering and Garrod, 2004). Lack of common ground regarding register expressions and data scarcity (e.g., for historical texts) could affect replicability and predictability. When variability between individuals in register use is high, then averages of language behavior across individuals may differ substantially across studies, reducing replicability. For instance, when asking participants to rate example stimuli that pertain to specific register (e.g., informal language variants), one would typically compute average ratings of the stimuli across many individuals. If individuals come from different geographic areas, or linguistic or social backgrounds, as one might expect with random sampling, their perception of stimuli may differ, leading to substantial variability in the computed averages^11^. Likewise, when examining conversational exchanges in a spoken corpus, aspects of the pronunciation and choice of words can provide insight into register. However, other aspects of each individual's linguistic and social experience may also affect phonetic realization, lexical, and morphosyntactic choices.

We need to disentangle individual language experience and use (as a “baseline”) from situation-specific aspects of language use [see also (Biber, 2009a), on the discussion of design issues in quantitative corpus linguistics methods]. To this end, research within the collaborative research center explicitly considers not only register use dependent on the situation but also associated inter-individual variation. This is done with different methods depending on the specific type of data and the associated research questions. For instance, “language situations” have been proposed as a useful method for assessing situation-dependent variability across speakers in contemporary language use (Wiese, 2020, see Section 2.3). For historical texts where we have little to no access to the situational context and different speakers, we explore inter-individual variation on the basis of switches between speakers within a narrative text, arguing that the narrator uses a different register than protagonists of the narrative. In lab experiments, we use within-participant designs or vary the role (e.g., interviewer vs. interviewee) of the language user, and collect meta-data on participant characteristics.

#### 1.3.3. Cognitive modeling of register

A theoretical challenge concerns the cognitive modeling of language register representations in relation to a general model of linguistic knowledge. Such modeling could provide constraints on hypotheses about register use and processing. Influential language theory research has—since the mid 1960s—contributed seminal insights into knowledge representations (Chomsky, 1965, 1986, 1995) and so has much research in cognitive (e.g., Lakoff and Johnson, 1980; Lakoff, 1986, 1990; Jackendoff, 1990, 1997, 2002; Bergen et al., 2003; Zwaan, 2004) and neuro-linguistics (e.g., Rizzolatti and Arbib, 1998; Pulvermüller, 1999; Hauk et al., 2004).

These cognitive approaches to language theory focused on standard language, and less on situational-functional language variability; by contrast, text linguistics (e.g., Van Dijk, 1972; Brown and Fraser, 1979; Irvine, 1979; Chafe, 1982; Halliday, 1988, as cited in Biber, 2019b) and quantitative sociolinguistics, examined situation-specific language use, with recent proposals conceptualizing language variability in a continuous situational space (Biber et al., 2020). Often, idealizations that abstract away from key factors affecting language use were used for modeling the mental representations implicated in language production, perception, and comprehension. Indeed, many factors driving register variation are not yet part of current models of grammar and of conceptual mental representations^12^. Likewise, in psycho- and neurolinguistics, research into real-time language processing in context has for a long time focused on the processing of standard language in the population, and the latter was equated implicitly with 18–31 year-old students in formal lab situations. But over the past two decades, investigations have also begun to broaden out to other language user groups among them children and adolescents (Schwab et al., 2021), mid-age adults (Huettig and Janse, 2015), older adults (Federmeier and Kutas, 2005; Maquate and Knoeferle, 2021; Adli, 2022), illiterates (Mishra et al., 2012; Huettig et al., 2018), and second language learners (Osterhout et al., 2008; McLaughlin et al., 2010; Ito et al., 2018). Drawing on these and other insights from psycholinguistic research, accounts of situated language processing have begun to include language user characteristics to model the observed inter-individual variability (Jannedy and Weirich, 2014; Münster and Knoeferle, 2018; Weirich et al., 2020). Related to the focus on inter-individual variation, much about mental representations remains to be uncovered regarding situation-dependent linguistic variability within one and the same group/language user and at the intersection with inter-individual variation in language use. For cognitively-oriented linguistics and psycholinguistics, how processing varies with subtle contextual changes like the degree of situational formality has not yet been widely modeled.

Within the collaborative research center on register, linguistic description and modeling goes beyond standard language and includes the mental representations of variants and their alternatives (see Section 4). In this endeavor, we also draw on insights into language variation and change gained from bi- and multi-lingual language users (e.g., Alexiadou, 2017; Wiese, 2021; see also Kroch, 2001; Adger, 2006 for a discussion of inter- and intra-individual variability).

### 1.4. Summary

In this article, we present an overview of methods (corpus studies, statistical/theoretical modeling of language register, and experiments in the field, the lab, and online) with a specific focus on illustrating the pervasiveness of situation-dependent register use across different languages, modalities, time periods, and cultures. In doing so, we focus on replication in an area of language use in which variability in individual language experience and use is likely high^13^. Building on the presented methods, we explore the consequences our findings have for a cognitive model of register knowledge. As a collaborative research center uniting scholars from different sub-disciplines of linguistics and the social sciences, our longer-term goal is to integrate the findings of the current 4-year funding phase into a more general model of register knowledge covering register change, learning, perception, comprehension, and production across different languages.While Sections 2 and 3 describe the project methods and results in depth, an overview can be found in Table 1.

## 2. Corpus-based approaches

### 2.1. Register classification, correlation, SOLT, and multi-modal approaches

#### 2.1.1. Contemporary and historic text corpora: German, old high German, and Swedish

In the CRC 1412 “Register”, we use a range of methods for analyzing and uncovering the pervasiveness of registers and their markers. Some projects use annotations of grammatical features and correlation analyses. For instance, an investigation into metaphor and metonymy (project A01 see Footnote 1) is compiling a corpus of contemporary German texts balanced for features of register variation which have proven relevant for metaphor and metonymy (see Goatly, 1994, 2011; Steen et al., 2010). The corpus comprises five subcorpora, speeches from the German parliament, news commentaries, sermons, light fiction, and debates from the competitions of the German debating society “Jugend debattiert”. Features like vertical and horizontal distance of interlocutors as well as literality vs. orality or persuasiveness were introduced in Systemic-Functional Grammar (Halliday and Hasan, 1985) and in Biber-style analyses (Biber and Conrad, 2009). Relevant properties of metaphors describe their form, conventionalization, and their content (literal and intended meaning of metaphors). Annotation guidelines ensure good inter-rater agreement. For the interpretation of the results of the annotation, we correlate the annotated properties of the metaphors and metonymies with features of the registers. First results show a clear interdependence between metaphor and register: Non-conventionalized and extended metaphors show up predominantly in commentaries and sermons, suggesting these metaphors occur predominantly in highly persuasive registers. This tendency is weaker in debates, which might be due to the time pressure of oral discourse. The expectation that oral discourse has a lower degree of metaphoricity than literal discourse throughout could not be confirmed. This suggests that previous very low counts of metaphoricity for oral discourse (as in Steen et al., 2010) might be related to the conversational nature of these data. Our data suggest a low degree of metaphoricity for fiction, in line with the results by Steen et al. (2010). Sermons conveyed the highest degree of register marking among the text types in the corpus with a high degree of non-conventional and extended metaphors.

In a further project (B04, Old High German and Swedish), we are correlating linguistic features with the function of “instruction” (imperative, subjunctive mood, and modal verbs) with situational variables like social role relationship (i.e., if the social relation between addressor and addressee is directed upwards, downwards, or equal). Significant correlations are interpreted as indicators for register-sensitive linguistic behavior. We found an effect of social role relationships on the choice of subjunctives (upwards) and modal verbs (downwards), indicating that social role constitutes a relevant parameter of register choice also in the earliest attested stages of the two languages.

While project B04 focused on historical languages and social role relationships, in project A05, we have validated the use of a measure of one dimension of register, namely formality, derived from two public corpora of Contemporary German. For the validation, we relied on annotations for levels of formality in the German standard dictionary Dudenredaktion (2015). The measure that we validated was the SOLT. We defined the SOLT of a German word as the log-frequency ratio of the rate of occurrence of a word in a corpus of written language with that of the same word in a corpus closer to oral language (i.e., movie subtitles). For example, the SOLT value of 1.09 for the word *Salon* (“salon”) indicates that *Salon* is twice as frequent in the written language corpus as it is in the subtitle corpus. Project A05 uses the SOLT measure as a proxy for the degree of formality of an expression. To validate this use, Sauerland (2022) reports that the SOLT value corresponds significantly with the formality level as it was set by the dictionary. Having the additional SOLT measure for formality and other dimensions of register enriches the methods portfolio for register. It is of course important to also understand the relations between the measures. Specially for formality, we already use naive speaker judgments in project C03 as measurement (see Table 1). Comparing these to the corpus-based and the judgment-based measures will further strengthen our array of methods.

#### 2.1.2. Ancient Egyptian texts: Multi-modal methods

**Figure 1:**
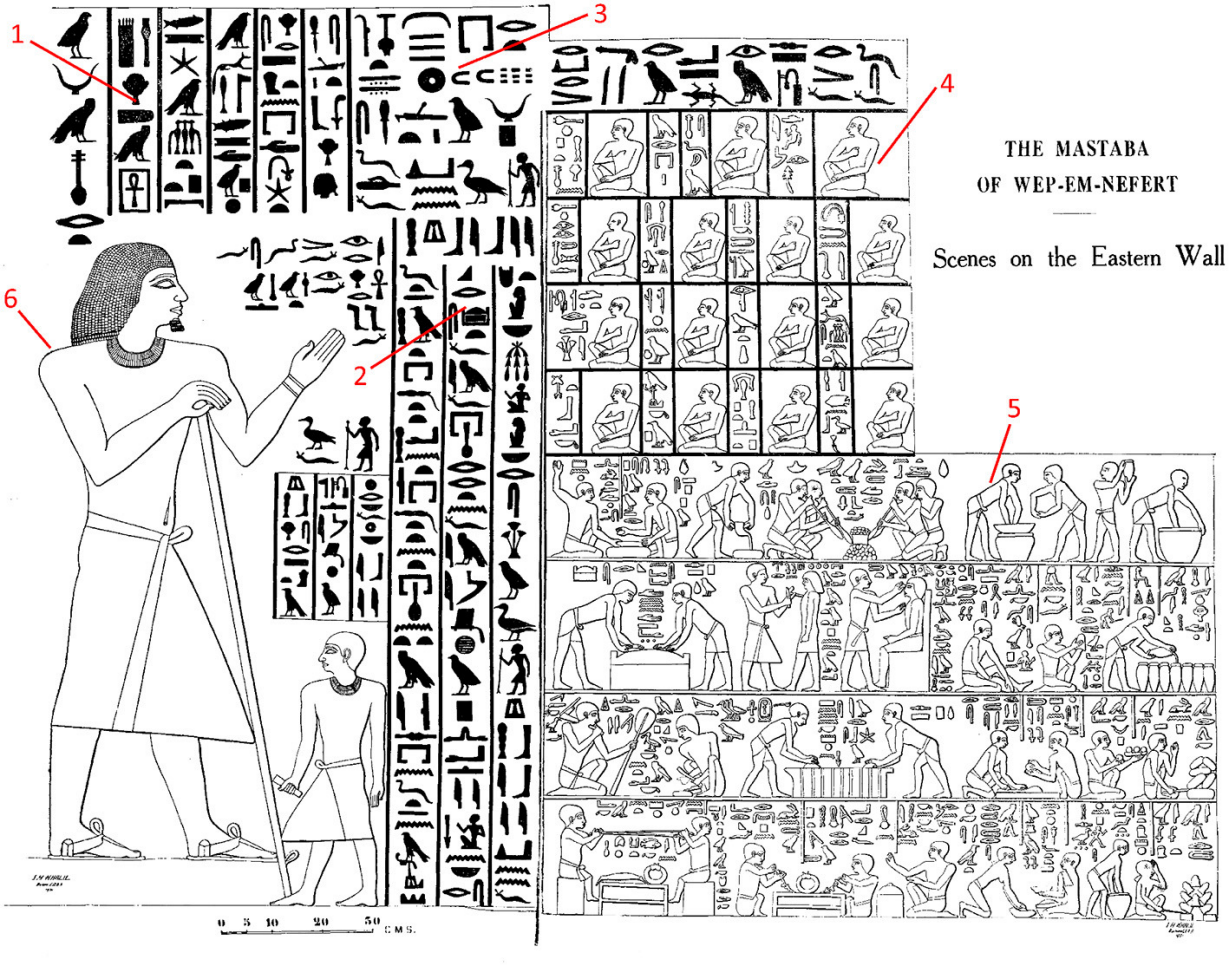
Project B03: Mastaba of *Wep-em-neferet* (G 8882), Relief of Eastern Wall, from Hassan (1936, Vol. 2, p. 190).

Our research further revealed the emergence of register in ancient Egyptian texts (B03). We approached the segmentation of Egyptian texts according to types of situations, speech constellation, and register-related characteristics, by considering linguistic as well as graphic features, such as the layout of the texts, the visual saliency of elements, color, and typography. These features are semiotically relevant with respect to information packaging, register-related text segmentation, and genre (e.g., Kress and van Leeuwen, 1996; Bateman, 2008). Since a large number of genres in Ancient Egypt includes pictures, a broader notion of “text” is applied that refers not just to textual sources but also to text-image compositions. Multimodal approaches (such as Kress and van Leeuwen, 1996) based on Halliday's Social Semiotics/Systemic Functional Grammar (Halliday, 1978; Halliday and Matthiessen, 2014) are deployed to examine the semiotic interaction of the different modes used in Egyptian texts (Kutscher, 2020). Furthermore, the extent to which pictorial aspects of multimodal compositions are relevant for register variation is of interest. Our research forges bridges between linguistics and the study of iconographic variation within and across text types, and the possible interdependency of iconographic as well as linguistic features in graphic communicational registers. For illustration, (see Figure 1) presenting a private tomb relief of *Wep-em-neferet*, a high official, and his eldest son *Iby* from the 3^*rd*^ millennium BC situated in the necropolis of Giza (G 8882). The composition of written and pictorial elements represents a testamentary disposition of *Wep-em-neferet* to his son. In addition to the pictorial representation of these two protagonists, there are details like their names and social function (see 1), the testament text (see 2), the date of the decree (see 3), a list of witnesses present (see 4), and representations of the manufacture of products to function as grave goods (see 5).

In Figure 1, both the text elements and the images are divided into several graphic zones (i.e., text segments) by means of frame lines, alignment, different scaling, and changes in the orientation of the reading direction. These graphic zones correspond to register-related linguistic features (e.g., more formal speech in the body of the testament vs. more informal speech in the dialogues of the workers in the grave product scenes). In addition, register-related differences concerning social roles are also expressed via depiction. For instance, figures representing high elite officials are depicted in a body posture expressing authority (see 6), in contrast to the more natural depiction of the socially low-ranked workers (see 5) or the special greeting gesture of the socially middle-ranked witnesses (see 4).

#### 2.1.3. Register classification by situational-functional criteria: Contemporary German and Persian

In addition to using correlation analyses, we developed a system to classify corpus texts according to situational-functional criteria via which a register estimation can be performed (see Table 2). Project A06 is currently using these classifications to research the register sensitivity of linguistic phenomena such as right-dislocations and referential expressions in existing corpora from German and Persian. Due to the nature of the data, we cannot distinguish with certainty between intra- and inter-individual variation. However, we assume that if one of two alternating constructions is preferred in a clearly distinguishable situational context, it is highly likely that the construction is truly register-sensitive. The text classes resulting from this classification were then cross-referenced with the very pervasive context indicator honorific use, which can be applied in both German and Persian. The German data showed that honorific use and speaker relation as well as the extent of being public coincide.

**Table 2:** Project A06: Classification and coding of pre-existing conversation data from the FOLK corpus (Schmidt, 2016) and the Pfeffer-Korpus (Pfeffer and Lohnes, 1984) on the basis of available metadata.

**Realm**	**Situational description**	**Category**
Media	Face-to-face, broadcasted	Face-to-face
	Audio-co-presence, phone call	Audio-only
Speaker	Non-acquainted speakers	Non-acquainted
relation	Acquainted or intimate speakers	Acquainted
Domain	Education, politics, gov. agency, interprofess. communication, etc.	Non-private
	Private contexts	Private
Size	Two-speaker	Two
	Multiple-speaker	More than two

We are working on extending this work on the basis of existing concepts, e.g., Biber (1994) and the extensive research done on modeling situational context in the SFL tradition (see Wegener, 2011; Hasan, 2014), in order to operationalize culturally independent, general situational-functional categories with practical applications for register studies through incorporation in data descriptions and in corpus metadata. By using a taxonomic structure, i.e., top-level categories with multiple sub-levels, users will be able to zoom in on the characteristics as much as the information provided with a text allows while still retaining comparability with other texts with less available metadata.

### 2.2. Latent Dirichlet Allocation analysis of register: Evidence from German

Extending the work in Section 2.1, project A04 uses tuples of (i) collections of grammatical features and (ii) sets of situational-functional parameters from large unstructured collections of texts to uncover an unknown set of registers. The method is rooted in fundamental assumptions about the nature of grammars. Under a probabilistic view of language (e.g., Hay and Baayen, 2005; Bresnan, 2007; Bresnan and Hay, 2008; Kapatsinski, 2014), it is plausible to assume that register grammars are acquired as weighted connections between lexical and grammatical features on the one hand and types of situations or situational-functional parameters on the other hand. By repeated exposure to specific grammatical features in specific types of situations, language users learn to assign high probabilities to said features in those situations. The classification of the situation in which language users find themselves is likely also probabilistic based on relevant situational-functional parameters. These parameters, such as prestige, formality, hierarchy, or educational background can be combined in many (including unseen) ways to produce a large number not discretely separable situations and associated registers. A direct consequence is that both texts and oral communication can belong to several registers with different probabilities (Biber et al., 2020, also consider a partially probabilistic model of registers but do not spell out a formal model).

**Figure 2:**
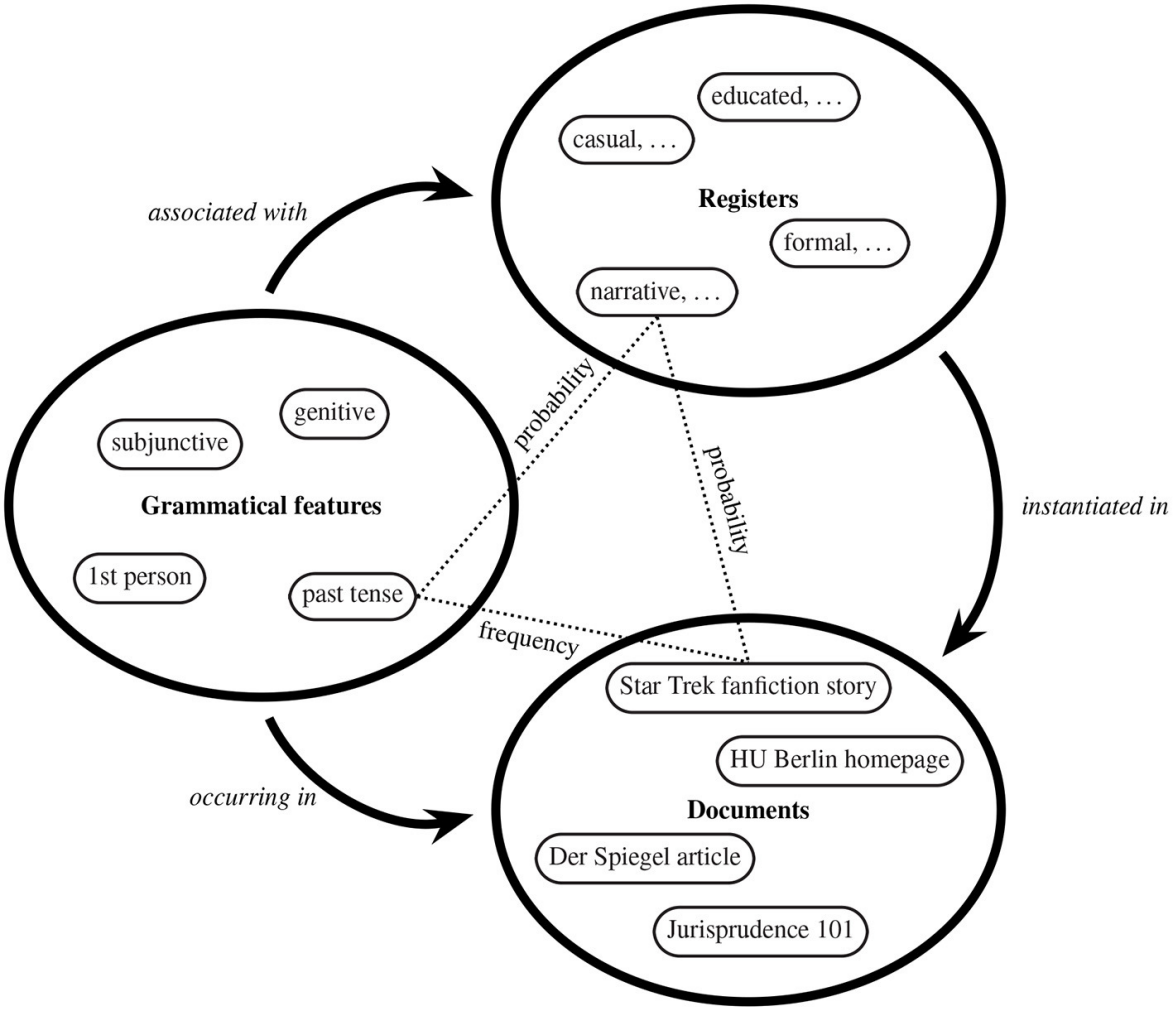
Project A04: Illustration of a probabilistic model of registers, grammatical features, and documents by examples; probabilities and frequencies are defined between each pair of elements from the respective sets.

The method is based on a fully-specified formal model of the relevant probabilities. It assumes that there is a set of registers, a set of grammatical features, and a set of documents. For the theoretical model, it is irrelevant whether the documents are just the documents in our corpus or the (rather fictional) population of “all documents written in the language”. The same goes for the features: They could be just the ones which we have annotated in our corpus, or “all grammatical features of the language”. Figure 2 illustrates the probabilistic mapping between these sets. Each grammatical category (such as the subjunctive or past tense) is instantiated with a given frequency in each document (for example an article in the German weekly news magazine *Der Spiegel* or a story from a website collecting fan fiction). By assumption, each register (for example an educated or a narrative register) is instantiated in each document with a certain probability, depending on how the writer classified the situational-functional parameters of the situation. In the example in Figure 2, the probability of educated registers being instantiated in a piece of fan fiction are probably quite low, but narrative registers have a high probability of being instantiated in such a story. Thus, our probabilistic view allows for the assumption of weighted mixtures of registers in a very natural way. Furthermore, each grammatical category arises with some probability in each register. It is important to note that we assume that these probabilities are defined between each pair of members of the respective sets, even if they are close to 0 in many cases. For example, the past tense might have a probability close to 0 in a register that does not require accounts of past events, but the fact that it is close to 0 is encoded in implementations of this model. It should be apparent that the frequencies and the two types of probabilities are not numerically independent of each other, and that the model imposes strong numerical constraints. Crucially, the set of registers and the probabilities are intrinsically unknown in corpora and, as we assume, in the language per se. We have to recover the unknown registers and the probabilities from the known features, documents, and frequencies obeying the numerical constraints imposed by our model. Certain Bayesian models are highly suitable for such tasks, and among these models is Latent Dirichlet Allocation (LDA, Blei et al., 2003; Blei, 2012), as popularized in topic modeling. However, LDA merely groups grammatical features and documents; it is thus unaware of the situational-functional parameters associated with the documents, which is why we call the uncovered features “potential registers” until further validation.

#### Outcome of LDA

**Figure 3:**
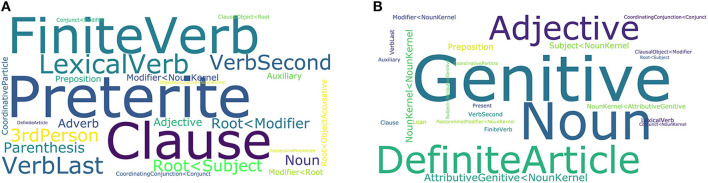
Project A04: Prominent grammatical features in two potential registers; **(A)** containing stories/accounts of events, **(B)** containing laws/texts on jurisprudence; font size corresponds to probabilities within the potential register.

The outcome of LDA is a pre-defined number of inferred potential registers and the probabilities with which each document and each grammatical category are associated with each potential register. We ran LDA on a subcorpus (22 million tokens in 2,475 documents) of the DECOW16B web corpus of German (Schäfer and Bildhauer, 2012), which has already been analyzed using LDA in the context of topic modeling (Bildhauer and Schäfer, 2016, 2017). We automatically extracted 1,631 grammatical features based on the rich linguistic annotation provided by the COW toolchain (Schäfer, 2015). We specified the algorithm to discover 25 potential registers. Both the prominent features and the top-ranked documents for many of the potential registers can be interpreted in terms of registers. For example, we find a potential register where the top documents contain stories and detailed accounts of events written in a predominantly lively tone with the prominent grammatical features as plotted in Figure 3A. The stories and detailed accounts are characterized by finite verbs in the past tense, complex clausal syntax, and a verbal style. Another potential register prominently contains laws and texts on jurisprudence. Its distinctive grammatical features are plotted in Figure 3B, and it is characterized grammatically by definite articles and markers of complex NP syntax (e.g., genitives, adjectives, noun–noun dependencies).

#### Additional manual annotation.

Register is clearly just one of many factors affecting the distribution of grammatical features in texts (others being, for example, style or content). To ensure that the purely grammatical (language-internal) distinctions found by the LDA as described above correspond to true register distinctions, we have developed a scheme (shown in Table 3) for annotating corpus documents for their elementary situational-functional parameters (similar to the one shown in Table 2). We assume these situational-functional parameters capture major distinction between the types of situations associated with registers as observable in web corpora like DECOW. Having both the LDA results and the manual annotation according to this scheme allows us to establish which of the potential registers found by the LDA are associated with situational-functional properties (and thus can be assumed to be true registers) and which are not.

**Table 3:** Project A04: Annotation categories.

**Parameter**	**Short illustration**
Education	Does the situation require an elevated educational background?
Interaction	Are there two or more interlocutors engaged in the conversation?
Proximity	Is the situation proximal and are there no strong politeness requirements?
Aim	What is the primary purpose of the communication? Possible categories are narration, joke, roleplay, reported dialogue, advertisement, instructions, default.

### 2.3. Analyzing register beyond grammatical features: Evidence from contemporary German, historic Germanic, and Egyptian

#### 2.3.1. Isolating intra-individual variability in historical texts

For historical contexts, it is particularly difficult to distinguish inter-individual from intra-individual variability. In project B04, we try to circumvent inter-individual variation by focusing on a single (group of) texts by a single author for Old High German and Old Swedish respectively: the Book of Psalms by Notker III of Saint Gall and the revelations of Saint Birgitta of Sweden^14^. In addition, this approach minimizes the influence of time, dialects, and genre. Most variation found within these texts should then be considered the influence of situational factors. On the one hand, narrative, argumentative, instructive, or dialogical passages alternate throughout these texts. On the other hand, numerous religious protagonists play a major role in both Notker's psalms and in Birgitta's revelations. These characters can often be positioned on a social hierarchy and it is reasonable to assume that Notker and Birgitta drew on their own register knowledge to devise these characters.

The differentiation between intra- and interindividual variation also is a challenge for Ancient Egyptian texts (B03). Texts were mostly written anonymously but producing a text involved at least four different roles: (i) a contracting authority ordering the composition of a text for a specific purpose, in combination with a selection/creation of pictorial representations accompanying it (e.g., the wall decoration of the monumental tomb); (ii) an author producing and conceptualizing the content, form, and style of the text-picture-entity; (iii) an editor compiling and collating written as well as pictorial resources; and (iv) a copyist merely reproducing an original. This diversity of participant roles within the process of text and picture production contributed to a shift of focus from producer to recipient. However, a stronger differentiation of recipient roles also seems to be required. To circumvent this problem, a group of texts from the corpus of Ancient Egyptian were selected which can be classified as “narrative” (i.e., the product of a narration process marked by the reporting of events in an iconic or consecutive manner). This is rendered by a text producer who actively regulates, creates, and frames the story as well as its formal and content-related aspects. Narrative texts are also characterized by their double- or multi-layered structure (Zeman, 2018) which means that information is communicated on the level of the narrator/text producer and the level of the protagonists (see project B04).

#### 2.3.2. Register shifts in contemporary German

For texts containing multiple registers occurring in sequence, we need to develop methods to identify such sequences (see Section 2.2 for a formal account of non-sequential register mixes). Project C04 develops methods to do so for contemporary texts written in academic registers by second-language learners of German. We investigate the abstract variable of noun modification (how a noun is modified). We identify all instances of noun modification by looking at all occurring structures that can be seen as variants of this variable. We used different layers of structural annotation, including dependency and morphological information (see Lukassek et al., 2021), accompanied by thorough manual processing. This strategy is genuinely variationist inasmuch as it attempts to provide an exhaustive account of all variants of a specific variable occurring in a corpus. In most quantitative register studies, a (sometimes tacit) assumption is that one text belongs to one register (see Biber, 1988, 2009a, 2012; Biber and Conrad, 2009). However, in several projects within the CRC (A04, C04, the B-projects) we observe that one text can include multiple registers (see also Egbert and Mahlberg, 2020). We use academic essays from our corpus of L2-authored texts (Falko, see Reznicek et al., 2012). Such texts instantiate an argumentative register. However, texts found in our corpus also contain narrative passages. We remain agnostic with respect to the function of these narrative passages within academic essays. Our primary goal at this stage is to identify them in a reproducible way without resorting to linguistic surface forms, as this strategy is problematic due to a lack of unique structural criteria characterizing narration exhaustively (see Zeman, 2018). One of the strategies we are pursuing is the probabilistic identification of narrative vs. non-narrative passages.

## 3. Experimental approaches: Isolating intra-individual variability

In experimental work (e.g., projects A05, A06, A07, and C projects), we conduct controlled laboratory and field experiments using contexts in which speakers/participants can infer their social relation to the interlocutor, the level of formality and(/)or other aspects of the situation. We assume that such information about the context influences lexical, morphosyntactic, and fine phonetic details related to register in language production (e.g., A05, A06, C02, C04, C05, C06, and C07) as well as lexical and compositional processes in language perception (A05, A07, C03, and C07). Inter-individual variability is controlled in some experiments. In others it is treated as random variability against which we can compare the systematic manipulation of formality in statistical models.

### 3.1. Examining register production via oral interviews, written elicitation, communicative exchanges, and “language situations”

#### 3.1.1. German, Persian, and Yucatec Mayan data from recordings in public vs. private settings

In project A06, we look at differences in linguistic behavior in contrasting situational settings from a cross-linguistic perspective. Complementing the corpus-based methods described in Section 2, we recorded conversational data in controlled situational settings^15^. The same participants were recorded in multiple conversations in which they were either acquainted or unacquainted with their confederate. The setting was a private room with comfortable furniture and decorations to encourage the participants to be relaxed and give them a sense of privacy. In a second set of recordings with the same participants, they were recorded in more public settings, once in an office talking to a university professor and once while driving in a taxi and engaging in a conversation with the driver. Here, the speakers were always unacquainted with each other. However, the levels of formality, expertise, and social prestige associated with profession of the confederate differed as perceived by the participants. Parallel data were collected for Persian in Tehran, German in Berlin, and Yucatec Maya in Felipe Carrillo Puerto (Yucatán, México). With this dataset, we look at how linguistic behavior differs intra-individually from situation to situation based on parameters such as location, acquaintance, and status while keeping the variation of the social categories minimal (similar educational backgrounds). The data will further allow us to look at register variation across languages and cultures, revealing which register parameters have a similar impact cross-linguistically and which ones may be more culture-specific.

#### 3.1.2. Register variation in fine phonetic detail of German in zoom-like conversations

Project C02 explores the effect of a controlled experimental setup on the expression of study participants by complementing this rich dataset with the study of variation in fine phonetic detail in experimentally controlled situations characterized by different levels of formality. We have defined formality in terms of the topic of a conversation as well as the social constellation between speaker and addressee. The social constellation was varied by means of the level of the perceived formality of the addressee which was experimentally established. The goal of our study was to investigate the controlled variation of the social constellation between speaker and addressee and the function of the discourse. For the latter, we selected themes where the speaker had to request something of the addressee (i.e., a deadline extension or a pay raise) while in the other situation, the speaker was to converse with their new neighbor by telling her something about the city they live in or a favorite restaurant. So, in one situation, the speaker was confronted with a face-threatening situation while in the other, there was no obvious gain or loss for the speaker.

For our experiment, we created a setup resembling a video conference: a monitor was placed at one end of the table and the participant was seated at the other end. Participants knew that they were interacting with a pre-recorded on-screen confederate^16^, yet they interacted naturally with the video (in fact, during the de-briefing period, speakers said that the situation felt rather natural to them, which in fact may be due to the experience with video conferences during the COVID-19 lockdown). Each participant saw the formal and the informal addressee and interacted with her accordingly. We have orthographically transcribed and phonetically semi-automatically annotated the time-aligned acoustic track. Labels were then hand-corrected. First data from over 30 participants from two northern German cities shows interesting differences between the different tasks and interlocutors, for example, a more variable and higher fundamental frequency and more dispersed vowels in the formal situation. We assume that an experience- or usage based probabilistic account of language (e.g., Barlow and Kemmer, 2000; Pierrehumbert, 2001; Wedel, 2006) with remembered instances of previously encountered speech forms linked to real-world experiences. Through exposure and the connection between speech forms and situations, native speakers assign higher or lower probabilities of specific usage forms with contexts.

#### 3.1.3. Namibian German: “Language situations”

Production of formal and informal registers is analyzed in project C07, too. We use data from Namibian German elicited by the “Language Situations” method (LangSit; Wiese, 2020), which is collected in the DNam corpus of German in Namibia (Zimmer et al., 2020). The LangSit method elicits naturalistic, ecologically valid, and controlled register-differentiated data, and it can be applied across different communicative situations, languages, speaker groups, and settings. Speakers are presented with a video showing, for instance, a traffic accident, and they then report the event to different addressees (e.g., a friend vs. a teacher). These two situations constitute an informal and a formal setting. This allows us to identify systematic differences under different levels of formality as truly intra-individual variation (see also A06, A07, and C05 among others). In the analysis, it turns out that non-standard lexical variants are distinct indicators of an informal register, while non-standard grammatical variants can appear in both the formal and the informal register. However, their frequencies differ across registers (Wiese et al., 2022).

#### 3.1.4. Development of productive register variation in German bachelor students

Project C05 contributes to the register research methods in the field of adults' late language development. It explores the register flexibility of students (mainly native speakers of German) recruited from a bachelor program for primary school teachers in relation to their acquisition of the language for the specific purposes of linguistics as an academic register. Register flexibility is understood as an individual productive skill which pertains to the capacity of the speaker for fast and controlled adjustments of language use based on sensitivity for changing communicative goals and circumstances (Kaplan and Berman, 2015; Qin and Uccelli, 2020).

Methodologically, the project combines written elicitation tasks with a grammar test (TEDS-LT) evaluating the development of linguistic terminology and declarative grammar knowledge (Bremerich-Vos et al., 2011). The test battery is complemented by standardized psychological questionnaires assessing personality traits (Danner et al., 2016), empathy (Paulus, 2009), and motivation (Thomas et al., 2018), to tease apart intra- from inter-individual variation and to control the students' aptitude for situated variation in academic communication. The project employs a longitudinal design applying the same test battery at three time points spanning the course of bachelor studies—at the beginning and end of linguistic courses and before. Each participant writes four explanations to grammatical issues. Two communicative contexts (e-mails to a pupil and to a fellow foreign student) instantiate forms of informal personal communication and are associated with colloquial registers. The other two contexts (a task in a tutoring class and in a linguistic exam) involve an institutional setting and require a more formal, academic language use. Much like in other projects, the elicitation task manipulates parameters of the communicative contexts, such as formality and familiarity. The task is thus suitable to test predictions from theories of register development as pragmatic entrenchment of linguistic behavior in holistically represented situational categories (Schmid, 2020). The contexts also feature specific combinations of situational parameters (social relation to the addressee and institutional setting) salient in early and later phases of register development. This setup targets the effects of fine-grained situational properties on the use of scientific or colloquial registers within the framework of Systemic Functional Grammar (Halliday and Matthiessen, 2014, see also A01).

The texts (*N* = 320) produced by the participants at the first testing point (first year students) were annotated on several levels to obtain form and meaning-based variables as indicators of register flexibility and metalinguistic knowledge (e.g., frequency of correctly and incorrectly used linguistic terms). In terms of data analyses, C05 combines quantitative and qualitative methods (i.e., accuracy evaluation of the explanations) to account for intra-individual and group-level variability in relation to conceptual development and metalinguistic awareness. The construct of register flexibility was operationalized as degrees of dissimilarity between the texts in relation to the four eliciting contexts. It was quantitatively assessed as the differences in the frequency of occurrence of register-sensitive linguistic features (Qin and Uccelli, 2020). Mixed-model analyses based on the frequencies of three lexical (grammar terms, discourse connectives, and stance markers) and three syntactic features (adverbial, relative, and passive clauses) revealed a general formality cline between explanations elicited in communicative situations appraised as personal communication versus as public academic activities. Stance markers and discourse connectives showed high situational selectivity in the informal contexts only; their frequencies varied significantly in explanations provided to a schoolboy or a to a fellow foreign student. The patterns of register variation found in the explanations of first year L1-students (see Figure 6) can be consistently interpreted with regard to the participants' varying degrees of familiarity with the academic institutions (school; university) and the communicative tasks (email, exam, tutorial) taken to reflect the pragmatic associations of linguistic behavior with the representation of situational categories. At the first testing point, participants exhibited higher register flexibility in the contexts of personal communication, adapting their linguistic choices not only to the institutional setting but also to their represented social relation with the addressee.

**Figure 4:**
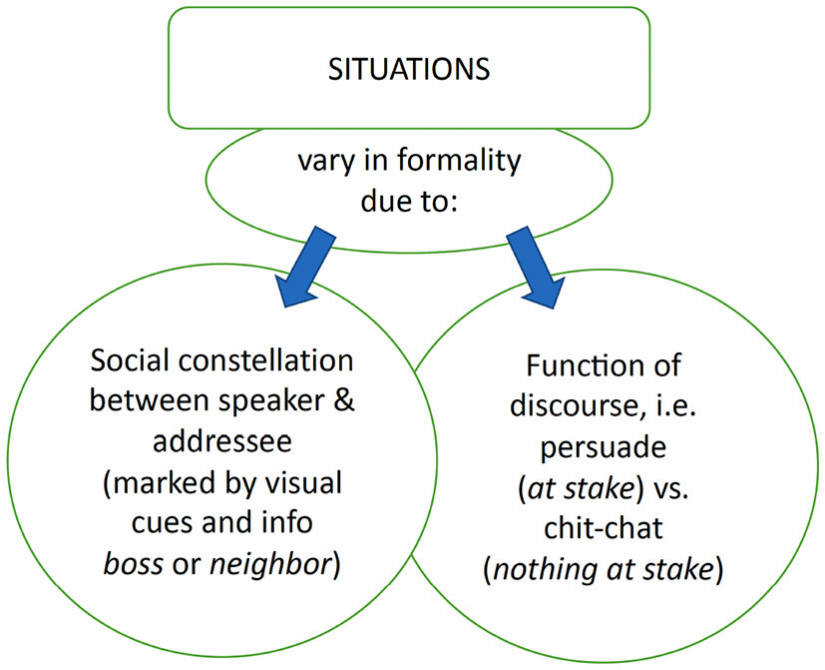
Project C02: Schematic representation of factors considered within a situation varying in formality.

**Figure 5:**
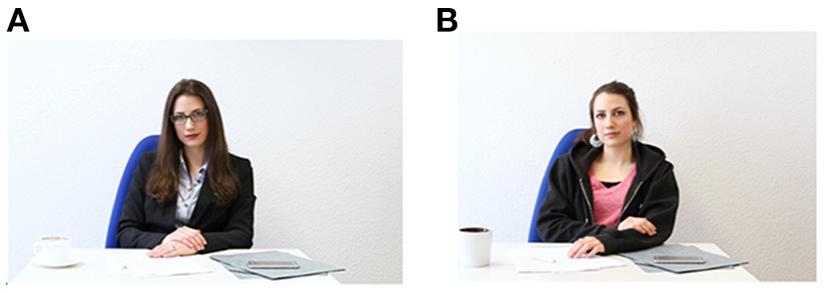
Project C02. (**A)** Formal confederate and **(B)** informal confederate.

**Figure 6:**
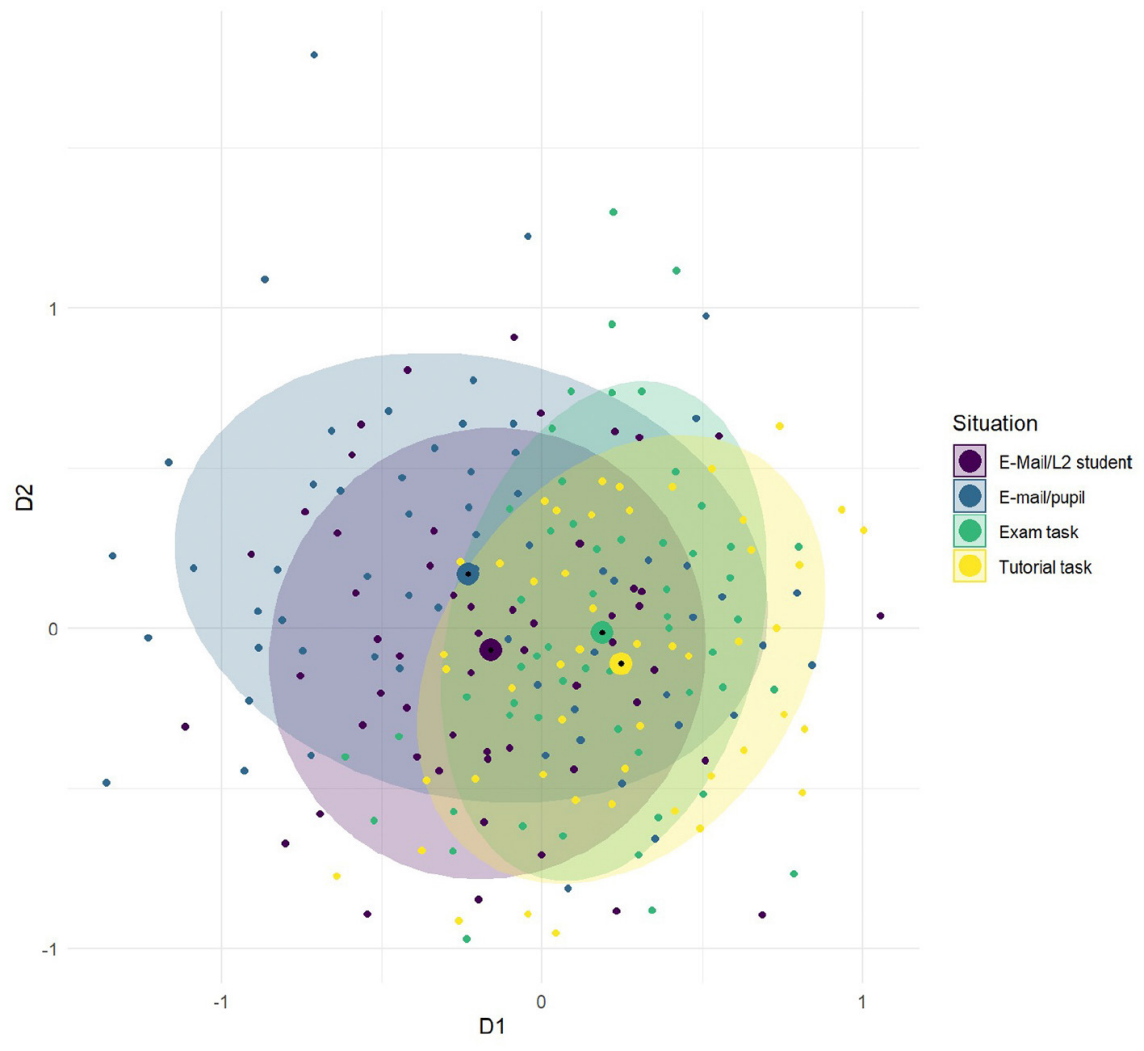
Project C05: Multidimensional scaling plot of a two-dimensional solution. D1—institutional setting (school, university); D2—social relation to the addressee. The observations (explanations) are colored according to the eliciting contexts. Ellipses were added to support visualization of the proximity relations.

#### 3.1.5. Written production of German temporal expressions in formal vs. informal contexts

**Figure 7:**
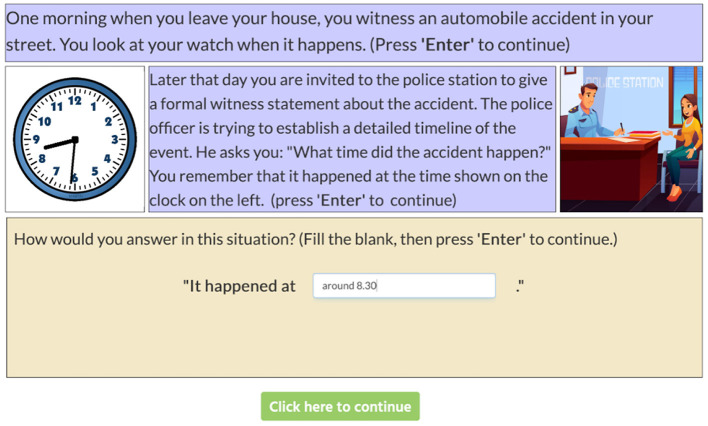
Project A05: Screenshot from production experiment—police station context (image from freepik.com).

Project A05 investigates the production of precise vs. imprecise numerical expressions—for example, whether an event is described as occurring at *8:31, 8:30*, or *about 8:30*—as a case study of intra-individual variation involving alternatives that differ in their core semantic content (Lavandera, 1978). Such cases are interesting because situational parameters beyond formality are expected to play a role in a speaker's choice between alternatives. In an internet-based production experiment, participants read a scenario in which they had witnessed an automobile accident and were subsequently asked what time it occurred; the time was displayed visually (see Figure 7). Seven information states (times) were tested, in two contexts, a police station (predicted to yield a high proportion of precise answers) and a party (predicted to yield a higher proportion of rounded and approximate answers). To make the task as natural as possible, a single-item fully between subjects design was employed, allowing inferences to be made about intra-individual variation on the basis of differences between participant groups. The results confirmed the prediction regarding the difference in frequency of rounding between contexts. A probabilistic interactive game-theoretic model was then fitted to these results, demonstrating that the observed differences in speaker behavior between contexts can be attributed to a different prioritization of speaker goals, with accuracy having greater importance in the police context, and hearer-oriented simplification greater importance in the neighbor context.

#### 3.1.6. Task- and addressee-dependent L1-English, L2-German register production

Extending insights on the effects of formality, research in C06 examines short-term register shifts in production. We shift toward a different register in order to accommodate the presumed needs of the interlocutor^17^. The non-native addressee register (NNAR, Roche, 1998) belongs to the clear speech register and is characterized by louder and slower speech, hyperarticulation, usage of more restricted vocabulary, and less complex syntactic structures (Zuengler and Bent, 1991; Roche, 1998; Bradlow and Bent, 2002). Up to now for German this register has only been investigated for low-proficiency non-native speakers, often confounded with a power difference. In project C06, we employed an experimental design with two sessions per participant—one with a non-native confederate (L1 English) as interlocutor eliciting NNAR and another with a German native confederate serving as a basis for comparison. By keeping the experimental sessions otherwise identical and controlling for interlocutor age and gender (matching native and non-native confederate), we are able to reliably pinpoint the influence of the addressees' nativeness and minimize the influence of power imbalances and/or prestige of the non-native L1. The German L2 proficiency (mid vs. high proficiency) of non-native confederates is assessed through ratings of intelligibility and accentedness collected via online experiments and used as a co-variate in the statistical analysis. Due to the pandemic the mode of recording had to be adapted. Instead of seating two participants in a small sound booth we recorded them in two adjacent rooms (the phonetics laboratory and an office) with both microphones connected to the same preamplifier, one channel assigned to each speaker. The participants communicated via zoom on two tablets. Even though this has the drawback of a less natural situation, it has the advantage of complete source separation (see Offrede et al., 2021 for a discussion of methodological issues in multi-speaker experiments). When speakers overlap during conversations this often poses problems for automatic transcription and forced alignment tools. Preliminary results from 20 sessions indicate that speakers slow down and produce less filled pauses when speaking to a non-native vs. native speaker.

### 3.2. Examining register perception via matched and open guise, rating studies, and eye-tracking experiments

#### 3.2.1. Experiments on Namibian German and English

Complementing the insights from the production experiments, projects C07, A07, A05, and C03 examine perception, using correction tasks, rating studies, and online comprehension experiments. C07 examines perception of registers in Namibian German, using the “newspaper correction” and open guise methods. We used the “newspaper correction” method in order to investigate the acceptability of lexical and grammatical contact linguistic features in formal written registers. Under this method, stimuli are mock newspaper articles representing model texts written in a formal register (Kellermeier-Rehbein, 2016). Participants are asked to act as editors and correct unsuitable language use. Applying the method, we see which linguistic features participants pick out as “wrong” in the formal register while using them in informal register (Wiese et al., 2022). We also see which features they accept even though they are not part of standard German in Germany. Participants showed systematic differences in the correction of experimental items, suggesting that the method is suitable to assess differences in the salience of register markers/variables (e.g., syntactic vs. lexical variables) and their involvement in the development of a new formal register in a language-contact situation.

Another method in C07 was the open guise method. This method elicits reactions to speech samples of the same speaker that differ with respect to linguistic categories such as standard and non-standard dialect. The method, originally developed by Soukup (2013), elicits evaluations/attitudes toward speakers, extending the matched guise method (Lambert et al., 1965). Unlike the matched guise method, it explicitly reveals that the same speaker produced different speech samples. We modified this method for register studies, using speech samples differing with respect to grammatical vs. lexical variables identified in our corpus study on Namibian German and asked listeners to evaluate this in two different experiments: In one, they are asked to assess the interlocutor as a friend or a teacher, and in the second they are asked to assess the speaker along semantic differentials. The results from pilot studies show that listeners associate different speech samples/registers of the same speaker with different roles (i.e., friend vs. teacher) and different evaluations of the speaker (e.g., with respect to intelligence or sense of humor).

Project A05 complements these studies on the effects of speaker role with studies on how the choice of precision level in context affects perceptions of the speaker (Beltrama et al., 2022). In a series of internet-based studies, we extend the matched guise technique (Campbell-Kibler, 2010; Beltrama, 2018) to the investigation of register, by varying not only the linguistic forms tested (e.g., the trip to the airport takes 49/50/about 50 min) but also the situational contexts in which they are used. Four types of contexts were studied: a casual conversation between strangers; a relationship-building context such as a chat among new coworkers, a persuasive context such as selling a car; and a for-the-record context such as testifying in court. We find that overall, precise forms elicit higher ratings on attributes relating to Status (e.g., intelligent, confident), whereas approximate forms elicit higher ratings on attributes relating to Solidarity (likable, laidback), and lower ratings on those related to Anti-Solidarity (uptight). But these associations are modulated by the conversational setting, in particular the demands on descriptive precision placed by the context and the interlocutors' goals. In future work we plan to incorporate these findings into modeling of production behavior, by exploring such potential social meanings as factors in a speaker's choice between numerical forms in context.

**Figure 8:**
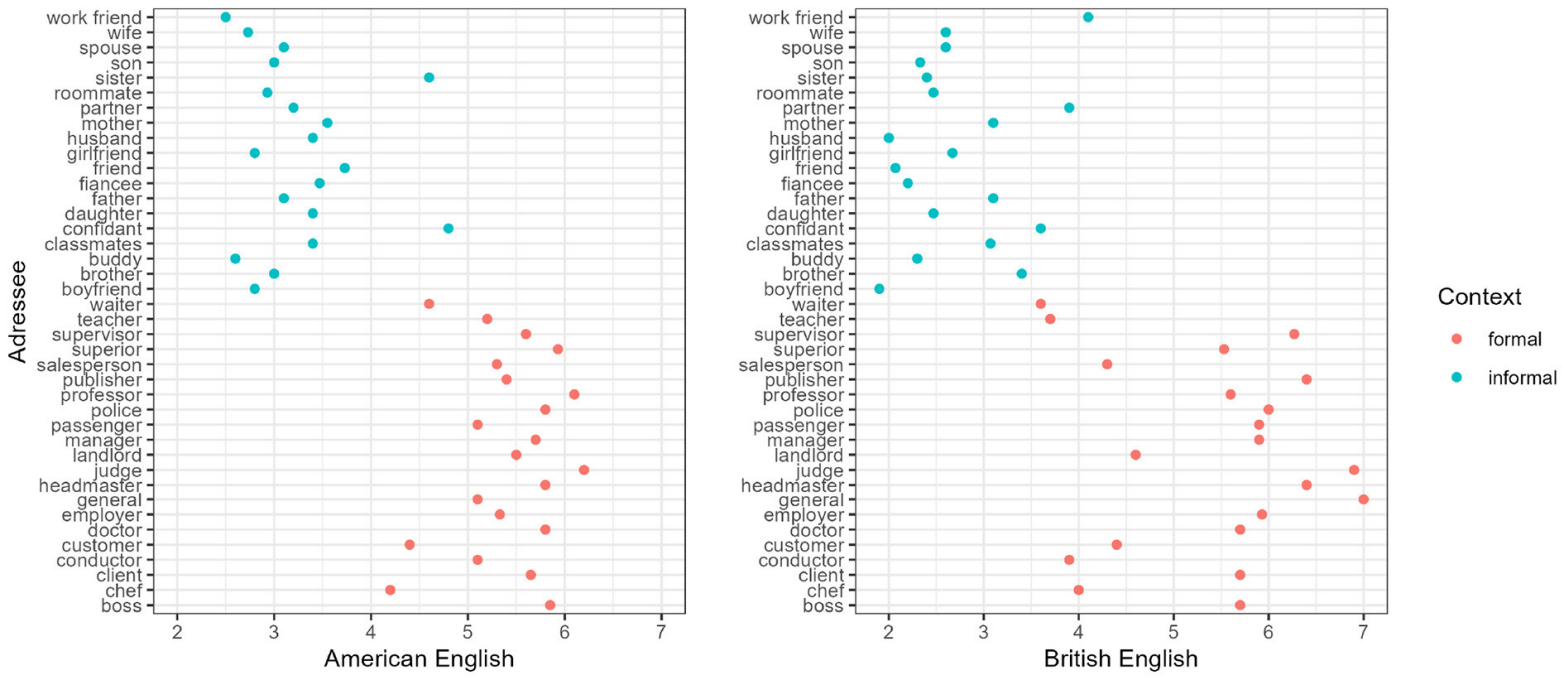
Project A07: Pre-test results of 40 formality manipulations using social relations of the interlocutors and 10 participants for American English and British English each. Participants read short stories, e.g., “*George Henderson works in a shop. The shop is deserted. He says to his manager/mother:”* and subsequently answered questions, e.g., “*Is George Henderson going to talk formally?”* on a 7-point Likert scale.

In Project A07, we further broaden the investigation of register effects to three sets of related phenomena in English dialects: negative concord (e.g., *I ain't seen nobody/anybody*.), pleonastic negation [e.g., *I miss (not) seeing you*.], and negative polarity items [NPIs, e.g., *John wouldn't (lift a finger to) help with the task*.]. These phenomena have been approached differently in theoretical linguistic (Horn, 1989), psycholinguistic (Dudschig et al., 2021), and sociolinguistic research (Labov, 1972; Eckert, 1989). Taking an integrated approach, we conducted the first set of experiments on negative concord, single negations, and NPIs in American and British English. As register use is known to be influenced by different situational and functional characteristics (Agha, 2006; Biber et al., 2020), in the main experiment, we created formal vs. informal contexts, manipulating social relations for hierarchy and familiarity (e.g., talking to one's *manager* vs. *mother*). Stimuli were validated through several pre-tests, including one for formality manipulations (see Figure 8). Pre-test results showed that our manipulation was overall valid, as suggested by a clear separation between formal vs. informal contexts. Some variability in the ratings emerged between American and British English, and overall across items. These results were used to inform the analysis of the main experiment, in which we assessed the appropriateness and interpretations of the aforementioned variants in relation to the formality of the context of use through a rating task. It is to note that while NPIs are part of Standard English, negative concord is dialectal and often considered as grammatically incorrect (Smith, 2001; Blanchette, 2017). Therefore, we adopted appropriateness ratings to prevent prescriptive judgments, which might be elicited by an acceptability rating task. We additionally assessed whether variability in such ratings was modulated by individual differences (e.g., dialect, age, gender^18^, and education). The preliminary results of this experiment suggest a register effect of negative concord in that it is perceived as less appropriate in formal than informal contexts, with no such effect for NPIs in formal vs. informal contexts (Rotter and Liu, 2022). We furthermore plan to investigate the pragmatic effects of the use of different variants (i.e., in relation to speaker perception and to social context), using the matched guise technique (Lambert et al., 1960; Campbell-Kibler, 2010; Burnett, 2019), as well as the register effects on linguistic behaviors of alignment or misalignment as signals of social distance (Brown and Levinson, 1987; Giles et al., 1991; see Pickering and Garrod, 2004, for considerations on processing).

#### 3.2.2. Eye-tracking register comprehension in German

**Figure 9:**
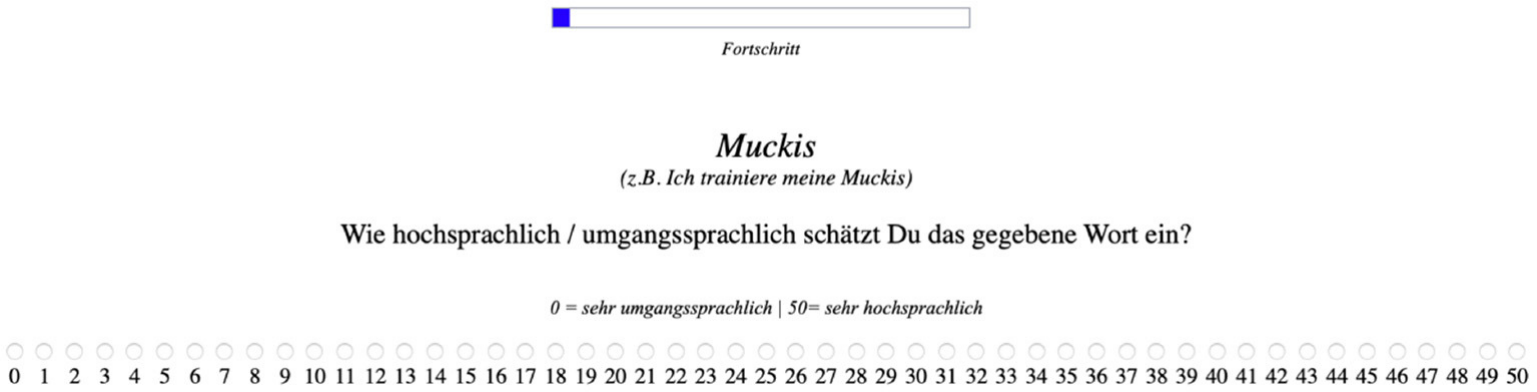
Project C03: Formality rating of a target word in a sentence context.

In C03, we complement the offline data (see Section 2) with real-time eye-tracking data on comprehension. We study the perception of register by manipulating the match between the level of formality of context sentences and the level of formality of object nouns in a spoken target sentence. In addition, we contrast such register matches with semantic congruence between a verb and its argument (e.g., matching “ties shoes” vs. mismatching “ties clothes”). To ensure validity, much work has gone into extensive pre-testing of the register manipulations while also assessing inter-indidivual variability. A 50-point scale ensured that the collected rating data were sufficiently fine-grained (see Figure 9). The lower end of the scale corresponds to low (“very informal”) and the upper end to high levels of formality (“very formal”). To reduce lexical ambiguity in some low-register words (e.g., *Mähne* can mean “horse mane” in formal language or “human hair” in colloquial language), individual words are shown together with a context sentence (see Figure 9). The language ratings showed clear register differences for 36 out of 40 critical items. Participants consistently rated words and sentences classified as “formal register” higher on the formality scale than their low-register counterparts. We took this to indicate that participants are aware of the register distinction. Eye-tracking results revealed first tentative insights into real-time register effects, too (Patarroyo et al., 2022).

We included a social-background questionnaire (participants' educational background, subjective social status report, and regional dialect) to explore inter-individual variability, which we hypothesized could modulate the ratings of high- and low-register variants. Statistical analyses performed using linear mixed-effects models revealed, for the sentence ratings, an effect of register, with high-register variants rated as more formal than lower-register ones. Furthermore, an interaction effect of register and dialect emerged: Dialect speakers rated high-register variants as less formal, and low-register variants as more formal, relative to standard German speakers. We found an effect of register on word formality ratings, with high-register variants rated as more formal than lower-register ones, as well as effects of social status and education, with words rated overall as less formal by participants with a higher (vs. lower) social status, and with a higher (vs. lower) level of education.

## 4. Mental representation of register

In addition to the methodological challenges highlighted in Sections 2, 2.3, and 3 (on uncovering registers and on isolating them given inter-individual variation), we are beginning to examine and model the mental representations implicated in the use and processing of register. Such investigation and modeling can further constrain, and help develop, testable hypotheses. For modeling, project C07 has looked at how register knowledge may be integrated into linguistic representations (see Wiese, 2021). Central to this approach is the notion of *communicative situations* (comm-sits) around which the linguistic system is organized and which can be characterized by situational characteristics, such as formality, mode, speaker constellation, and distance (Wiese, 2021, p. 5). Lexical entries in Wiese (2021) contain grammatical (e.g., phonological, syntactic, and semantic) information and information related to a comm-sit. In this way, a word like *Mauken* (“feet”) may be linked to a comm-sit representing situational settings in which German is spoken with friends, which in turn may be associated with an informal register.

Modeling in project A05 relies on iterated Bayesian models of speaker and hearer already in use in pragmatics (Frank and Goodman, 2012; Burnett, 2019). A05 is extending such techniques to model the interaction of register and semantic differences in case of a choice between a round and a precise numeral. Where such models are successful, they show that there are cases where registers variants coexist in the same language much like C04 observes coexisting registers within one and the same text type. Whether register shifts require extra attention is tested in another project (C06) by manipulating the cognitive load. The participants have to remember a dot pattern that is either irregular and therefore difficult to reproduce or that is a simple regular pattern. The assumption is that the register shift is not automatic and requires extra cognitive resources.

Complementing communicative situations and Bayesian approaches, we also use indices (inspired by Jackendoff, 2002) to model effects of register and/or context formality during real-time sentence comprehension. In project C03, much like in the other studies, we assumed that context (formality) matters for language variability (e.g., Adger, 2006) and assessed its effects on real-time language processing. We investigated, for instance, whether context formality-register congruence (Table 4 for design) affects sentence comprehension rapidly and modeled these congruence effects (**Figure 11**). Validity, given high inter-individual variability, is ensured via counterbalancing as illustrated in Table 4 (both versions of the context and of the target sentence contributed to each of two conditions).

**Table 4:** Project C03: Eye-tracking reading pilot.

**Condition**	**Linguistic context**	**Target sentence**	**English translation**
Register match	Formal	Der Detektiv düpierte_(*formal*)_ den Gauner.	The detective duped the villain.
Register mismatch	Informal	Der Detektiv düpierte_(*formal*)_ den Gauner.	The detective duped the villain.
Register match'	Informal	Der Detektiv übertölpelte_(*informal*)_ den Gauner.	The detective scammed the villain.
Register mismatch'	Formal	Der Detektiv übertölpelte_(*informal*)_ den Gauner.	The detective scammed the villain.

We varied formality and register of the context sentence such that it either matched or mismatched the register of the target sentence. The “-” sign indicates counterbalancing conditions.

**Figure 10:**
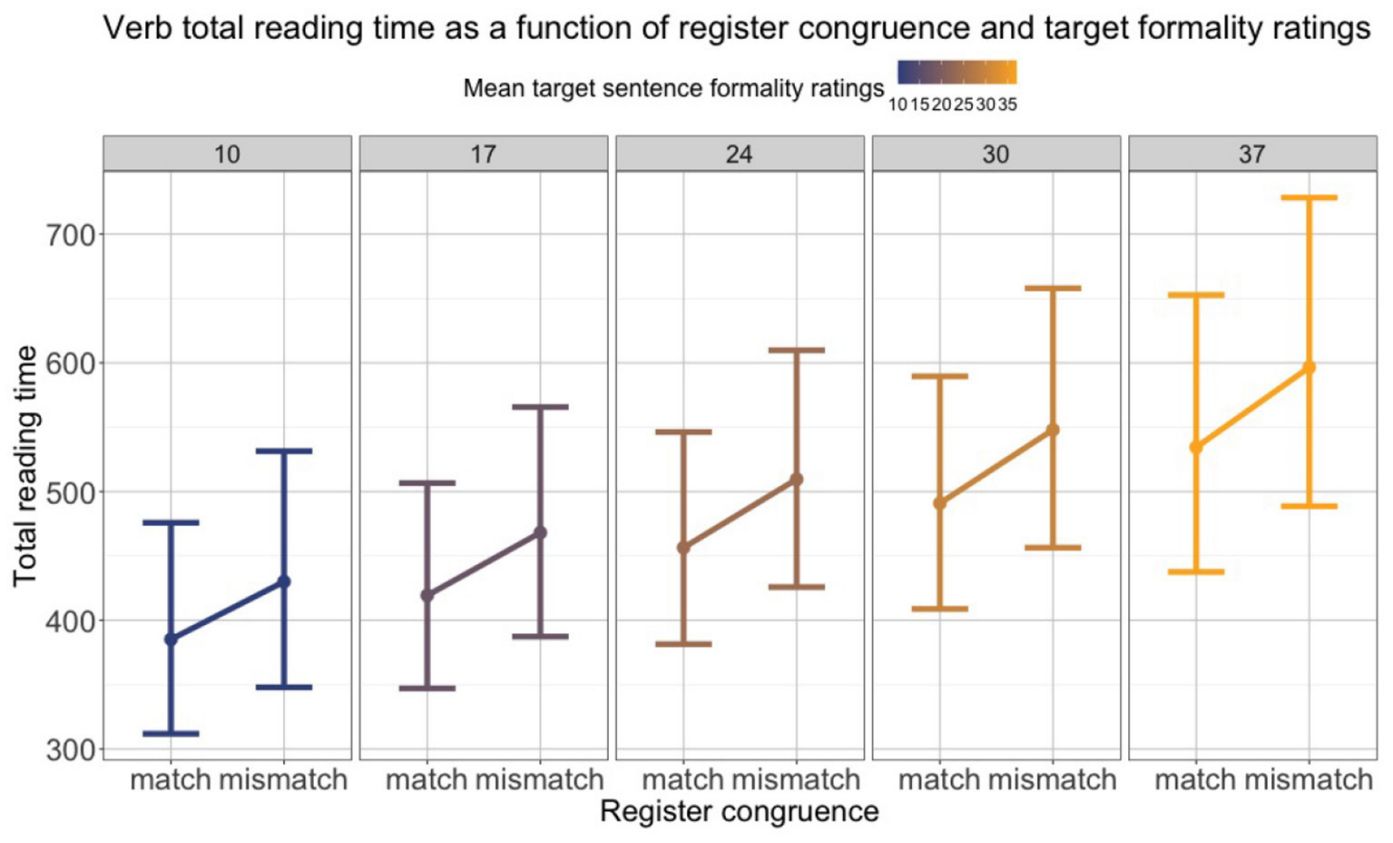
Project C03: Effects of register congruence and average target sentence formality ratings (obtained through an independent Web-based rating task, on a scale from 0 = very informal, to 50 = very formal) on target verb total reading time, in an eye-tracking pilot study (*N* = 8). Register-mismatching verbs yielded longer total reading times, relative to register-matching verbs. Furthermore, longer total reading times at the verb region were observed in target sentences with higher formality ratings, relative to those with lower formality ratings.

In an eye-tracking sentence reading pilot study in project C03, German adults read two context sentences followed by one target sentence (fillers interleaved; 3/4 of these were followed by yes/no comprehension questions as attention checks). We manipulated congruence of context formality and target sentence register (match vs. mismatch), and tracked eye movements during reading. Analyses performed on first-pass duration, regression path duration, and total reading times (see Rayner, 1998, for definitions) for the verb of the target sentence (Figure 10, *N*_*participants*_ = 8) revealed, as expected, longer total reading times for register mismatches compared to matches (effects in earlier measures at the verb n.s.). Exploratory post-hoc analyses further showed that total reading times were also longer for sentences with higher (vs. those with lower) formality ratings (Figure 10).

**Figure 11:**
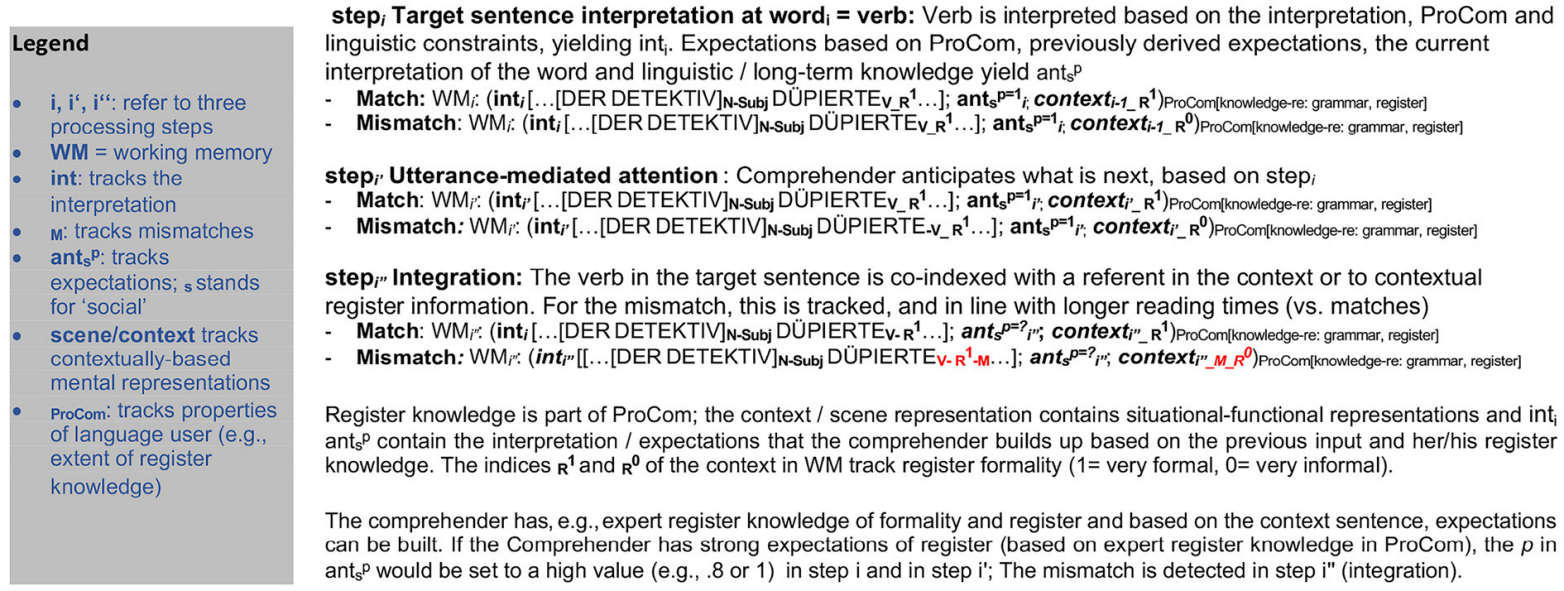
Figure C03: An example of the mental representations that may be formed upon interpreting the formal target sentence *Der Detektiv düpierte den Gauner* (“The detective duped the villain”), preceded by either a formal, register-matching or an informal, register-mismatching context.

The results suggest that comprehenders can swiftly integrate register information during online sentence processing. At issue was what mental representations language users may form when incrementally interpreting *Der Detektiv düpierte den Gauner* (“The detective duped the villain”), preceded by a matching formal context versus by a mismatching informal context (see Figure 11). Figure 11 illustrates how mental representations of the target sentence might be integrated with the formality/register of the context sentence as the verb “duped” (*düpierte*) is processed. In line with Münster and Knoeferle (2018), we assume three steps (sentence interpretation, language-mediated attention, and integration with context). Indices mark each step (see blue entries in Legend, Figure 11). Working memory (WM) representations track the unfolding interpretation (int) and expectations (ant); context representations track the representations of the context sentences and can be marked for formality and/or register. Effects of register mismatch are modeled at the third step, when the target sentence register is integrated with context representations of formality and register via co-indexing (yielding matching vs. mismatching representations, see Figure 11, notation in red font). The formality-match must be probabilistic to also capture effects of stimuli properties (e.g., of degrees of formality illustrated in Figure 10) and to offer a linking hypothesis from the model to reading times.

## 5. Summary and conclusions

The present article reviewed projects of the CRC 1412 “Register” that illustrate the pervasiveness and robustness of register phenomena. We observed register effects for both contemporary and historical texts, across many different languages and cultures, as well as speech and text, in production, learning, and perception/comprehension. In such a framework, register knowledge/use is a pan-human phenomenon. As Table 1 showcases (Column “Preliminary results”), we observed register effects not only in British and American English, but also in German, Old High German, Swedish, and Egyptian. Intra-individual variability is captured, permitting us to zoom in on situation-specific variability in language use, production, and perception/comprehension within an individual. Not only do register effects replicate in corpus research (A01, B04, B03, A06), they also show up in elicited production experiments (C02, C07, C05, and C06) and language use ratings (A07, C03, C06). These established methods provide insights into language use and production; we complemented them with methods like eye-tracking (C03), newspaper correction (C07), and open/matched guise (A05, C07). The latter revealed register effects on compositional processes in language comprehension, an area in which research on register remains somewhat scarce.

Based on the reported findings (Table 1), we can speculate about the emergence of register phenomena. Perhaps these result from social differentiation and/or hierarchization (e.g., elite vs. non-elite) lead to communicative situations with different degrees of formality (e.g., informal vs. formal and in systemic-functional linguistic terms elicited by proximity vs. distance respectively). These processes [at the historical level often accompanied by sedentarism (vs. nomadism), state formation, evolution of ideology and religion, etc.] manifest themselves in the situation-specific use of language, with interlocutors able to switch between varieties according to their distinct social roles and personae (which depend on situational-functional contexts). Following that logic, we can assume that most interlocutors can (consciously or subconsciously) recognize and/or use more than one register (for exceptions, see Footnote 17) and also acquire registers. Register is on that account intra-individual functional linguistic variation in a specific social setting; it does not (indexically) point to the identity of the user in a specific situation. The latter point may distinguish register variation from dialects or sociolects; it can be viewed as *usage*-based (in contrast to *user*-based).

The insight that register phenomena are pervasive is supported by annotations of text and correlation of contextual with linguistic features; by methods that can uncover register fully probabilistically (LDA); by a range of experimental paradigms including matched and open guise, language situations, and newspaper correction tasks. Offline measures like acceptability, appropriateness, and formality ratings are complemented by online measures that can provide insight into register perception in real time. We showed how existing and new corpus and experimental methods can be adapted to challenges imposed by the study of register phenomena. These may complement each other and be applied to different types of data. From the empirical observations, we are beginning to model the mental representations implicated in the processing of register as well as add register knowledge to existing Bayesian pragmatic models of rational language use to develop and constrain hypotheses. But the modeling of register and of the implicated mental representations is in its early stages—much remains to be done on this topic in future research within the CRC 1412.

## Data availability statement

The raw data supporting the conclusions of this article will be made available by the authors, without undue reservation.

## Ethics statement

The studies involving human participants were reviewed and approved by the Ethics Committee of the Deutsche Gesellschaft für Sprachwissenschaft (DGfS). The patients/participants provided their written informed consent to participate in this study. Written informed consent was obtained from the individual(s) for the publication of any potentially identifiable images or data included in this article.

## Author contributions

PK conceptualized the article, provided a first draft of the introduction, and integrated the input from the contributing projects. Each contributing project provided written input for the article. JL and DS revised the introduction. RS and VP revised the second and third section, PK and AS revised section 4. AL provided feedback on the revised version, PK and JL integrated the revisions and prepared the third version. All authors commented on the third version, PK, JL, VP, and DS prepared the final version. VP and AS created Table 1. DS provided a draft of the response to reviewers and PK edited the draft. SK, GoS, and DS contributed text on SFL. PK revised the manuscript text. VP and DS integrated the revisions and prepared the fourth version. All authors contributed to the article and approved the submitted version.

## Footnotes

^1^The CRC 1412 “Register: Language Users' Knowledge of Situational-Functional Variation” is a third-party funded institution that incorporates scholars from Humboldt-Universität zu Berlin, Leibniz-Centre General Linguistics, and Universität zu Köln working in different (sub-)disciplines on one common overarching subject. Projects within the center investigate different aspects of register using multiple methodological and theoretical approaches. Projects are organized in three subject areas A, B, and C and have numbers. When referring to studies and their results from particular projects in this paper, we use the shorthand project names A01, A02, ..., and C07. For further information on each of the participating sub-projects, see the web page https://sfb1412.hu-berlin.de/projects/. The research is funded by the Deutsche Forschungsgemeinschaft (DFG, German Research Foundation)—CRC 1412, 416591334.

^2^This also incorporates the intra-textual linguistic context, such as surrounding sentences, etc. in a given text.

^3^The most prominent are Field-Tenor-Mode (Halliday, 1978), Context Metafunction Resonance (Hasan, 1995), Generalized Structure Potential (Halliday and Hasan, 1985), Action-Relation-Content (Hasan, 2001), and System Networks (Hasan, 1984).

^4^Wegener (2011) presents an application of the SFL model to multimodal communication strategies in a medical context.

^5^The social relation between participants as a main situational feature encompassed by the term “tenor” is a complex construct comprising factors (Hasan, 1973; Halliday, 1978; Neumann, 2013) such as age, gender, ethnic, social, or religious origin as well as achieved status, education, and expertise, among others. It also includes relationships in a communicative interaction between individuals or groups.

^6^When using the term *informal* in the context of linking hypotheses, we mean hypotheses that are formulated in a non-mathematical manner. This usage of “formal” differs from the instances in the context of formal vs. informal registers.

^7^The reference to “situational contexts” draws on Hasan (2014, p. 9), Kytö (2019, p. 137), and on Biber and Conrad (2009, p. 6). The “situational context” indicates the communicative purpose, the channel, participants, and their relationships as well as topics.

^8^In more recent works, Biber et al. (2020) proposed refinements of MDA.

^9^“Text witness” is an established term from Historical Linguistics and the field of philology; it is often used in the context of textual criticism. “Textual criticism” aims to arrive at an understanding of the historical creation and transmission of texts.

^10^Additionally, the variant *Mauke(n)* has a specific, terminological use in the domain of equestrianism. Additional inter-individual factors can therefore stem from expert status in this domain.

^11^However, not all variation can be attributed to discernible factors. It cannot be excluded that a remainder of “noise” in the data has to be treated as random.

^12^The focus of research on language processing and use has differed across sub-fields of the language sciences. In sociolinguistics, considerable attention has been given historically to language use as a function of societal groups, among them groups based on gender (Tannen, 1991; Laws et al., 2017; Holmes, 2018), age (Eckert, 1997; Diessel, 2009; Lieven et al., 2009), and social class (Bernstein, 1971; Trudgill, 1972) (see also Labov, 1966/2006; Xiao and Tao, 2007; Eckert, 2012, 2018; Mather, 2012). From there, sociolinguistics has moved toward looking at language use in smaller local groups, and toward the function of language as a communicative device of a social group (“third wave” Eckert, 2012) (see Soukoup, 2018).

^13^Good scientific methods include making sure that once-observed behavior can be elicited again when someone else uses identical (“exact replication”) or similar (“conceptual replication”) methods. Concerns regarding replicability have been discussed in corpus research [see (Millar, 2009; Leech, 2011), on modal auxiliary verbs)], see https://corplingstats.wordpress.com/2017/02/16/the-replication-crisis/, and Biber (2009a), König (2010), and Lüdeling and Hirschmann (2015). Replicability has also been discussed for laboratory experiments on, for instance, context-based expectations in language processing (DeLong et al., 2017; Ito et al., 2017; Nieuwland et al., 2018) and the embodiment of language (Rommers et al., 2013; Zwaan, 2014).

^14^Notker (ca. 950–1022) was a monasterial magister, and Birgitta (ca. 1303–1373) was a member of Sweden's political and cultural elite. They wrote texts that represent two Germanic varieties, Old High German and Old Swedish, Sonderegger (1987) and Andersson (2014).

^15^In an extensive sociolinguistic survey, we collected information about the participants.

^16^To find suitable addressees we have created choreographed videos with the same female person with different attire and hair styles. From a set of six different pictures from these videos, the two were selected that contrast most on the factor formality when independent raters judged the different persona types or personalities on 15 attributes on a 7-point Likert scale. The persona in picture A (on the left of Figure 5) was judged to be the most formal persona while the other addressee (B on the right in the picture) was rated to be most informal. In addition, subjects were told they were speaking to either their boss (or a professor) or a neighbor (or a fellow student).

^17^Consider, however, that in language development, early stages may indeed be characterized by a single register. We may also see a single register in language users with severely limited language abilities. By contrast, highly skilled language users may shift from one register to the next with ease and without noticeable delays. While such variability likely exists, one may ask whether they should shape the definition of register. The answer might be affirmative when considering that the present research endeavor foregrounds the pervasiveness of register effects. But arguably such pervasiveness can occupy the non-extreme ranges of register variability (e.g., shifting between two registers). The latter binary shift is what the bulk of reported studies in the present manuscript focus on. We do not consider in the present article cases in which register and speaker differ like writers of legal contracts vs. writers of aircraft manuals.

^18^Our data were balanced for gender.
